# Topoisomerase I poison-triggered immune gene activation is markedly reduced in human small-cell lung cancers by impairment of the cGAS/STING pathway

**DOI:** 10.1038/s41416-022-01894-4

**Published:** 2022-07-06

**Authors:** Jessica Marinello, Andrea Arleo, Marco Russo, Maria Delcuratolo, Francesca Ciccarelli, Yves Pommier, Giovanni Capranico

**Affiliations:** 1 Department of Pharmacy and Biotechnology, Alma Mater Studiorum—University of Bologna, Bologna, Italy; 2 Cancer Systems Biology Laboratory, The Francis Crick Institute, London, UK; 3 School of Cancer and Pharmaceutical Sciences, King’s College London, London, UK; 4 Laboratory of Molecular Pharmacology and Developmental Therapeutics Branch, National Cancer Institute, NIH, Bethesda, MD USA

**Keywords:** Mechanism of action, Chemotherapy, Small-cell lung cancer, DNA damage response

## Abstract

**Background:**

Current immunotherapy strategies have contrasting clinical results in human lung cancer patients as small-cell lung cancers (SCLC) often show features of immunological cold tumours. Topoisomerase 1 (TOP1) poisons are effective antitumor drugs with good efficacy against lung cancers.

**Methods:**

We used molecular, genetic and bioinformatic approaches to determine the mechanism of micronuclei formation induced by two TOP1 poisons in different human cancer cells, including SCLC cell lines.

**Results:**

TOP1 poisons stimulate similar levels of micronuclei in all tested cell lines but downstream effects can vary markedly. TOP1 poisons increase micronuclei levels with a mechanism involving R-loops as overexpression of RNaseH1 markedly reduces or abolishes both H2AX phosphorylation and micronuclei formation. TOP1 poison-induced micronuclei activate the cGAS/STING pathway leading to increased expression of immune genes in HeLa cells, but not in human SCLC cell lines, mainly due to lack of STING and/or cGAS expression. Moreover, the expression of STING and antigen-presenting machinery genes is generally downregulated in patient tumours of human lung cancer datasets.

**Conclusions:**

Altogether, our data reveal an immune signalling mechanism activated by TOP1 poisons, which is often impaired in human SCLC tumours.

## Introduction

Among all forms of tumours, human lung cancer has a high incidence and is the leading cause of cancer deaths. Lung cancer is constituted by different diseases distinguishable based on genetics, molecular and biological features. Small-cell lung cancer (SCLC) is a disease characterised by inactivation of *Rb1* and *Tp53* tumour suppressor genes and activation of *MYC* and/or *SOX2* oncogenes thus supporting cell proliferation [1, 2]. SCLC is initially sensitive to chemotherapy and radiotherapy, which are current standard treatments, but recurrent tumours are often unresponsive to further treatments. Approved initial therapy includes cisplatin and etoposide or irinotecan, while only topotecan has been approved for relapsed SCLC.

The natural alkaloid camptothecin (CPT), its derivatives (topotecan and irinotecan) and synthetic indenoisoquinolines, are effective antitumor drugs which target DNA topoisomerase I (TOP1) [3–6]. CPT analogues are FDA-approved drugs for the standard therapeutic regimen of human lung, ovary and colon cancers, and indenoisoquinoline analogues are in early phases of clinical trials (NCT01794104 and NCT03030417 at ClinicalTrials.gov). Their antitumor efficacy is likely due to a relatively high preferential cytotoxicity against proliferating cancer vs. normal cells. TOP1 poisons selectively target TOP1 in living cells by forming transient DNA-TOP1 cleavage complexes (TOP1ccs) leading to an increase of transcription/replication conflicts, R-loop formation, DNA damage accumulation, cell apoptosis and genome instability [3, 7, 8]. Interestingly, poisoning of TOP1 by CPT can increase mitotic errors and micronuclei [9–11]. Micronuclei are portions of chromatin or chromosomal fragments that are excluded from main nuclei and enclosed by nuclear membranes. Micronuclei frequency is generally increased in cancer cells as compared to normal cells, and is considered a marker of genome instability. Micronuclei were shown to mediate the activation of type I Interferon (IFN) and IFN-stimulated genes (ISG) in cancer cells through the cGAS-STING signalling pathway [12, 13]. However, the underlying mechanism of micronuclei formation triggered by TOP1 poisons is not fully established.

Human SCLCs are not highly sensitive to checkpoint immune inhibitors even though modulation of antitumor immunity remains an active investigation field. DNA-damaging agents are still at the front line of patient treatments and new ways to use them in the chemotherapy of SCLC are highly needed [2]. TOP1 poisons cause DNA damage and replication stress which can be exploited in new combinations of synergistic agents [14]. In addition, DNA-damaging agents and radiation can activate innate immune genes in cancer cells which could trigger an effective antitumor immunity in patients [13, 15–17]. However, the elicited mechanisms by which TOP1 poisons kill cancer cells are not fully understood. Here, we have uncovered aspects of micronuclei formation by TOP1 poisons in cancer cells and the molecular defects of human SCLC that largely impair innate immune gene activation by TOP1 poisons. The findings highlight genetic and molecular features of human SCLC that can be exploited in patient stratification improving precision medicine strategies.

## Materials and methods

### Cell lines and treatment

The cancer cell lines HeLa (RRID:CVCL_0030) and U2OS (RRID:CVCL_0042) were purchased from ATCC (LGC Standards S.r.l., Milan, Italy). SCLC cell lines H209, H889 and DMS114 have been kindly provided by Anish Thomas (NCI, NIH) [18]. An U2OS-derived cell line stably overexpressing RNaseH1 was obtained and cultured as reported already [19]. For culture conditions, see Supplementary Information. Exponentially growing cells were exposed to TOP1 poisons for the indicated time and concentrations. In the case of co-treatments, cells were pre-incubated with flavopiridol (1 μM) or MG132 (25 μM) or H151 (2 µM) for 1 h before the addition of TOP1 poisons. 5’-Azacitydine (5 µM) was administered 48 h before TOP1 poisons addition. Detailed procedures are provided in Supplementary Information.

### Immunofluorescence microscopy assay

Purification of S9.6 antibody was performed as previously published [19] and detailed in Supplementary Information. S9.6 and γH2AX (S139-phosphorylated H2AX) staining was performed as previously published [19] and detailed in Supplementary Information. cGAS and STING staining was performed as previously published [17] and detailed in Supplementary Information. To measure micronuclei levels, adherent cells were stained as previously reported [17] and detailed in Supplementary Information. Cells in suspension were treated at cell density around 7 × 10^4^ cell/mL with the same protocol but, 48 h after the end of treatments, 2 × 10^5^ cells were cytospinned using Cytospin 4 (Thermo Shandon, Runcorn, UK) onto a 26- × 76-mm glass slide and then fixed as for adherent cells.

### RNA extraction, retrotranscription and qRT-PCR

Total RNA was extracted and retrotranscribed as previously reported [17] and detailed in Supplementary Information. We tested our cDNAs for a panel of cytokines; cDNA was amplified in Biorad CFX Connect Real-Time System by using SsoAdvanced Universal SYBR Green Supermix (#1725274, Bio-Rad, Hercules, CA, USA) and a set of validated primers from each gene locus (Bio-Rad; complete list in Supplementary Table S1). The amplification protocol was set according to the manufacturer’s instructions. Specificity of PCR products was routinely controlled by melting curve analysis and agarose gel electrophoresis. ΔΔCt comparison method was used in order to calculate genes fold change and each gene expression was normalised on CytB.

### STING gene silencing

HeLa cells were transfected with Lipofectamine RNAImax Transfection Reagent (Invitrogen, Thermo Fisher Scientific, Waltham, MA, USA) and 20 nM siRNA against STING (Ambion siRNA #1 128591, Ambion siRNA #2 128592). The protein expression level was monitored against time from 24 to 96 h post transfection by western blot. All drug treatments were performed 48 h after transfection and cells were re-transfected with siRNA against Sting after drug removal (72 h after first silencing). Western blots were performed as previously reported [17] and detailed in Supplementary Information.

### RNaseH1 and STING overexpression

Cells were transfected with 2.5 µg/well of plasmid (pRH1 for RNaseH1, gently furnished by F. Chedin, University of California, DAVIS; NET23 pEGFP-N2-1174 for STING, #62037 Addgene, Watertown, MA; USA), and 5 µL/well of Lipofectamine 2000 (Invitrogen, Thermo Fisher Scientific, Waltham, MA, USA). Detailed procedures are reported in Supplementary Information. Western blots were performed as previously reported [17] and detailed in Supplementary Information.

### Determination of cellular cGAMP

cGAMP levels were quantified as previously reported [17] and detailed in Supplementary Information.

### Bioinformatic analyses

Data about copy number variations (CNVs), mutations and gene expression were processed and maintained by the Ciccarelli group at The School of Cancer Studies of King’s College London and The Francis Crick Institute, as part of the Network of Cancer Genes Database [20]. Computation of mutation rates, correlation between gene expression and enrichment scores and histogram plotting were performed using tidyverse (RRID:SCR_019186), GSVA library (RRID:SCR_021058) and R base scripts as detailed in Supplementary Information. Dataset sources, harmonisation of gene expression data and data analysis are specified in Supplementary Information. Code used in the bioinformatic analysis is available at https://github.com/marcrusso/Marinello_et_al_2022.

## Results

### TOP1 poisons enhance micronuclei formation mediated by nuclear R-loops and DNA damage

**Fig. 1:**
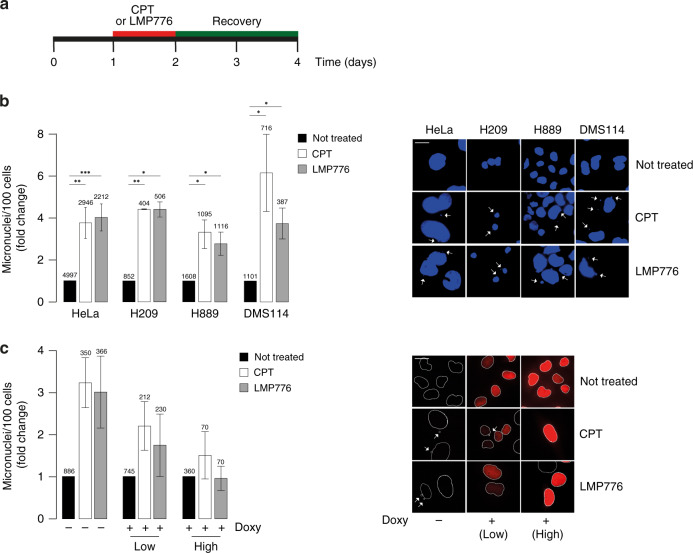
Induction of micronuclei by CPT and LMP776 is similar in human HeLa and SCLC H209, H889 and DMS114 cell lines, and is reduced by RNaseH1 overexpression. **a** Schematic representation of experimental conditions. One day after seeding, cells were treated for 24 h with TOP1 poisons and, after drug removal, let to recover for 48 h. **b** Cancer cells were treated for 24 h with 100 nM CPT or 200 nM LMP776 and then recovered for 48 h in a drug-free medium. Micronuclei were then stained with DAPI and counted with fluorescence imaging. Bars represent treated/control ratios and show mean values ± SEM (biological replicates for each cell line are: five for HeLa, two for H209, three for H889, and three for DMS114). Numbers of analysed cells are reported above the bar for each sample. Representative IF images are reported on the right. Asterisks indicate statistical significance in comparison with untreated cells by *t* test. *P < 0.05, **P < 0.01, ***P < 0.001**. c** Micronuclei were counted 48 h after release from drug treatment (100 nM CPT or 200 nM LMP776 for 24 h) in U2OS_RH cells, which are stably transfected with doxycycline-dependent RNaseH1-expressing vector. U2OS_RH cells were then exposed to doxycycline (+doxy) or not (−doxy) for 48 h before treatment with TOP1 poisons. RNaseH1 expression was detected with an antibody against a FLAG tag fused to the enzyme. RNaseH1-expressing cells were split into two categories (low and high) depending on the level of RNaseH1 expression. Representative IF images are reported on the right. Statistical significance of three biological replicates calculated with *t* test with respect to no Doxy are: CPT low *P* = 0.054, CPT high *P* = 0.068, LMP776 low *P* = 0.191, LMP776 high *P* = 0.058.

As TOP1 poisoning by CPT has been shown to lead to micronuclei formation [11], we have determined the levels of micronuclei in human SCLC (H209, H889 and DMS114) and HeLa cells treated with low doses of Top1 poisons (CPT and LMP776) and released from drug treatment for 48 h (Fig. 1a). In particular, we treated cancer cells for 24 h with 100 and 200 nM concentrations of CPT and LMP776, respectively, as they induced similar levels of micronuclei in HeLa cells (Fig. 1b). Micronuclei were present in untreated cells at very similar levels in all the studied cell lines (about 3–4 micronuclei/100 cells, Supplementary Fig. S1), and CPT and LMP776 increased three- to six-fold the amount of micronuclei in SCLC cells with slight differences (Fig. 1b and Supplementary Fig. S1). H889 cells showed the lowest increase (three-fold). However, only modest differences were detected among all cell lines (Fig. 1b). Different effects were observed for CPT and LMP776 in DMS114 cells only, but they were not statistically significant (Fig. 1b). Thus, the findings indicate that micronuclei formation by TOP1 poisons is likely a consistent phenomenon in many cancer cells, and that no marked different effects of CPT and LMP776 were detected in HeLa vs. SCLC cells.

To understand mechanistic aspects linking TOP1 poisoning to micronuclei formation, we determined whether R-loops could have a role in mediating micronuclei formation. To test this possibility, we used a U2OS-derived (U2OS_RH) cell line stably overexpressing a human FLAG-tagged RNaseH1 under a doxycycline-inducible promoter [19]. Levels of RNaseH1 overexpression in doxycycline-induced U2OS_RH cells were determined with immunofluorescence microscopy (IF) using an anti-FLAG antibody. Micronuclei induced by both CPT and LMP776 were reduced in doxycycline-treated cells in comparison with untreated cells (Fig. 1c). Interestingly, micronuclei reduction was marked or intermediate in U2OS_RH cells expressing RNaseH1 at high or low levels, respectively (Fig. 1c), suggesting a dose-dependent effect of RNaseH1 expression. Thus, RNaseH1 overexpression markedly reverted the effects of TOP1 poisons on micronuclei formation (Fig. 1c). RNaseH1 itself increased basal micronuclei levels in untreated U2OS_RH cells (Supplementary Fig. S2), as reported already [19], likely due to the functional roles of DNA:RNA hybrids in DSB repair mechanisms [21].

**Fig. 2:**
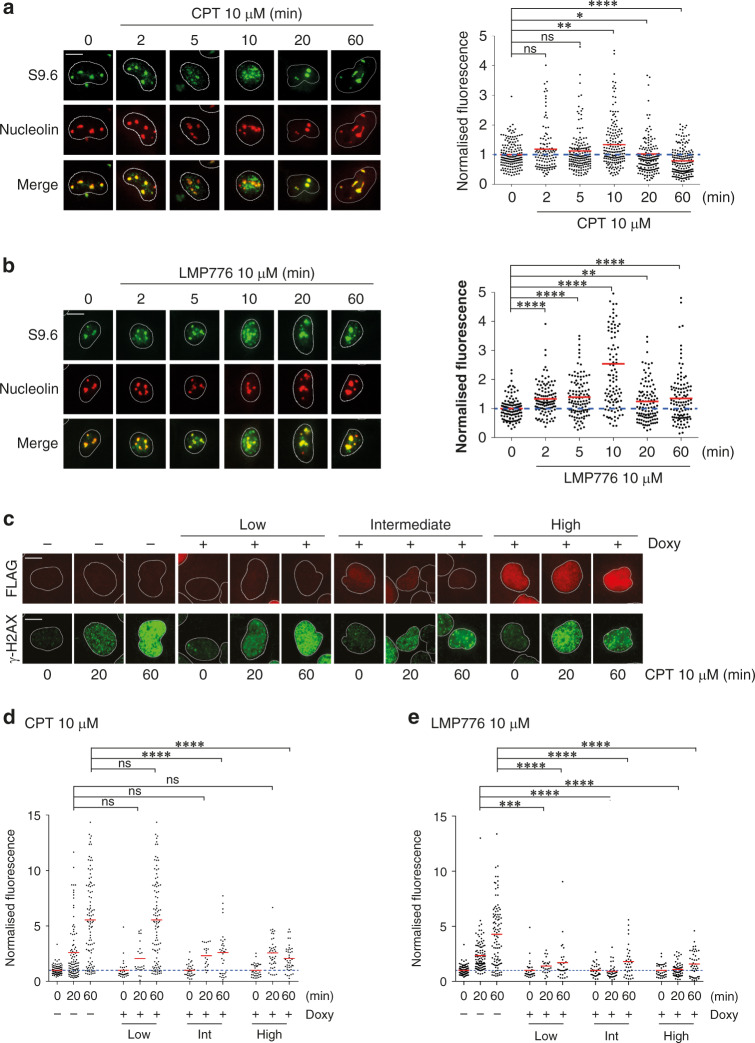
TOP1 poisons increase nuclear R-loops and induce R-loop-mediated γH2AX foci in HeLa cells. Cells were treated for indicated times with CPT (**a**) or LMP776 (**b**) and nuclear R-loops were detected by co-staining with DAPI (blue), anti-hybrid S9.6 (green) and anti-nucleolin (red) antibodies. Quantitative plots on the right represent nucleoplasmic S9.6 fluorescence normalised over untreated cells. Dots show single-cell values and red bars are means for each sample. Asterisks indicate statistical significance in comparison with untreated cells by the Kolmogorov–Smirnov test. *P < 0.05, **P < 0.01, ***P < 0.001, ****P < 0.0001**. c** Immunofluorescence analysis of U2OS_RH cells to detect γH2AX foci (Ser139 phosphorylation, green) and RNaseH1 expression (red) after the indicated times. **d**, **e** nuclear γH2AX levels in U2OS_RH cells treated with CPT or LMP776, respectively. Cells exposed to doxycycline were split into three groups with low, intermediate or high RNaseH1 expression levels, respectively (low, intermediate and high). Asterisks indicate statistical significance in comparison with untreated cells by the Mann–Whitney test. *P < 0.05, **P < 0.01, ***P < 0.001, ****P < 0.0001. Scale bars: 10 μm.

Next, we wondered whether the TOP1 poisons could trigger an increase of R-loops in cells. We showed previously that CPT has a biphasic effect on nuclear R-loop levels, which is TOP1 dependent in HCT-116 colon cancer cells [22]. Therefore, we directly determined the kinetics of R-loop alterations by IF experiments in HeLa cells treated with the two TOP1 poisons by performing double staining with S9.6 (Ab against DNA:RNA hybrids, [23] and an antibody against nucleolin, which stains nucleoli [19]. The results showed an increased hybrid signal in the nucleoplasm but not in nucleoli of cells treated for a few minutes to 1 h with the TOP1 poisons (Fig. 2). LMP776 was able to increase R-loops levels at somewhat higher levels and for longer times than CPT (Fig. 2a, b), in agreement with the knowledge that the half-life of TOP1ccs is longer for LMP776 than for CPT [24]. As R-loops are mainly co-transcriptional [25], we also determined the effects of transcription inhibition and found that the CPT-induced increase of R-loops was dependent on ongoing transcription (Supplementary Fig. S3). The hybrid specificity of S9.6 has recently been shown [26] and we checked its specificity under our experimental conditions with different experimental approaches. The highly-pure S9.6 antibody (Supplementary Fig. S4A, purity about 80% of the total preparation) recognised RNaseH1-sensitive nuclear structures in U2OS_RH cells (Supplementary Fig. S4B), as nucleoplasmic S9.6 signals were fully abolished by RNaseH1 overexpression (Supplementary Fig. S4C, D). As a further control, the antimetabolite methotrexate could not increase nucleoplasmic S9.6 signals in HeLa cells (Supplementary Fig. S5). Thus, the data overall show that the levels of DNA:RNA hybrids are induced in a time-dependent manner by the studied TOP1 poisons in HeLa cancer cells.

As genome instability can derive from DNA damage, we then wondered whether the observed increase of R-loops can mediate DNA damage induced by CPT and LMP776 by determining the level of S139-phosphorylated histone H2AX (γH2AX, a marker of DNA cleavage) at short times after drug exposure of U2OS cells overexpressing RNaseH1. As above, we could clearly detect doxycycline-induced cells having different levels of RNaseH1 by splitting the cells in three groups with low, intermediate and high RNaseH1 expression (Fig. 2c). We found that RNaseH1 expression reduced and almost abolished CPT and LMP776-induced γH2AX foci in a clear dose-dependent manner (Fig. 2c–e). These results demonstrate that TOP1 poison-induced DNA damage is mediated by R-loops in agreement with other studies [27, 28].

Taken together, our findings demonstrate that TOP1 poisoning by CPT and LMP776 can induce micronuclei in the studied human cancer cells at similar levels, and that R-loops play a mechanistic role in micronuclei formation and DNA damage induced by TOP1 poisons.

### Micronuclei induced by TOP1 poisons can activate the cGAS/STING pathway leading to immune gene activation

**Fig. 3:**
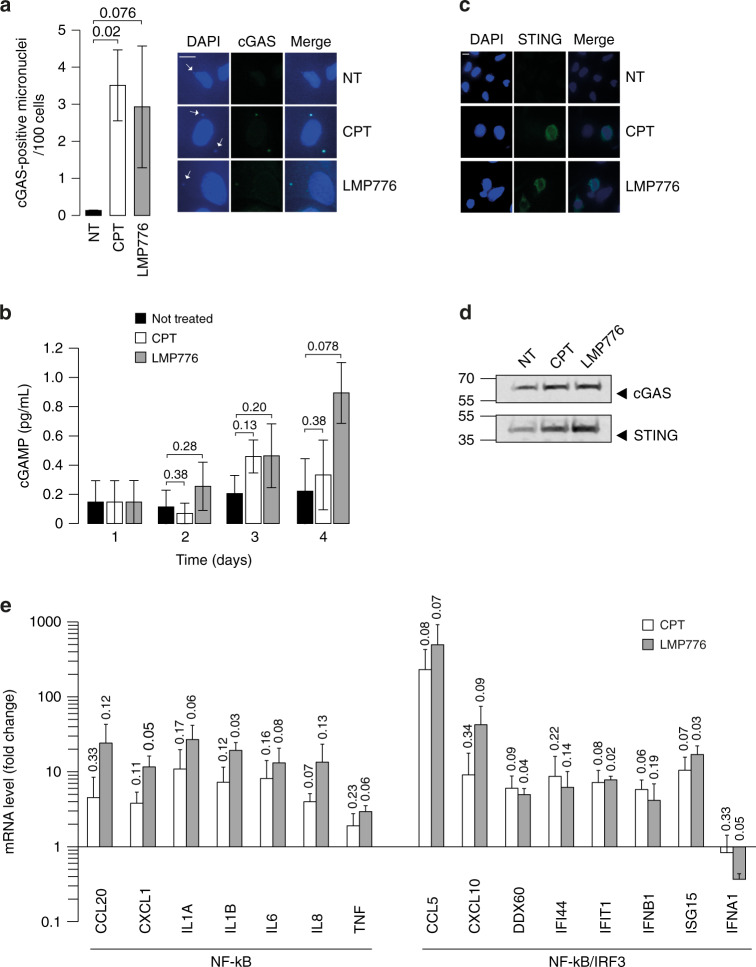
In HeLa cells, TOP1 poisons induce micronuclei formation dependently on the cGAS/STING pathway. **a** cGAS recruitment in micronuclei after TOP1 poisons treatment was determined by immunofluorescence assay. Quantitation data of cGAS-positive micronuclei per 100 cells and representative images are reported in the panel. Scale bars: 10 μm (one-tailed ratio paired *t* test). **b** cGAMP quantitation by ELISA assay in non-treated or treated cells following the experimental schedule presented in Fig. 1a. Two biological replicates have been performed (one-tailed ratio unpaired *t* test). **c** STING activation after TOP1 poisons treatment was determined by immunofluorescence assay. Representative images are reported in the panel. Scale bars: 10 μm. **d** Expression levels of STING and cGAS proteins were determined by WB in HeLa cells at time 4 (Fig. 1a). Red Ponceau is reported in Supplementary Fig. 6. **e** mRNA level determined by qRT-PCR of a selected panel of cytokines in HeLa cells after treatment with TOP1 poisons (time 4 in Fig. 1a). Expression levels are indicated as fold change over non-treated cells. *P* values are calculated in comparison with untreated cells and are reported as asterisks or numbers above bars (one-tailed ratio paired *t* test).

Next, as TOP1 poisons were able to increase micronuclei in cancer cells, we asked whether the cGAS/STING signalling cascade was activated by micronuclei leading to innate immune gene expression, as previously reported in SV40 expressing cells [29] and cancer cells [30]. First, we determined the recruitment of cGAS to micronuclei with IF microscopy and co-staining with DAPI and cGAS-specific Ab, which allowed us to calculate the fraction of cGAS-positive and -negative micronuclei for each condition (Fig. 3a). The data showed that the amount of cGAS-positive micronuclei per 100 cells significantly increased from 0.13 to 3.50 and 2.93 for CPT and LMP776, respectively (Fig. 3), suggesting that cGAS was recruited to micronuclei in the cytoplasm of cells treated with TOP1 poisons (cGAS-positive fraction of 3% untreated cells versus 30% and 20% for CPT and LMP776, respectively). To expand these results, we determined the time course of cGAMP levels in HeLa cells exposed to CPT or LMP776 with an ELISA assay. The results showed that cGAMP levels increased during cell recovery up to 48 h after drug removal in treated cells but not in untreated control cells (Fig. 3b). LMP776 induced somewhat higher cGAMP levels than CPT (Fig. 3b). These results show that CPT- and LMP776-triggered micronuclei can recruit and activate cGAS leading to an increase of the signalling molecule cGAMP.

As cGAMP acts as a second messenger activating STING on the endoplasmic reticulum (ER) membranes [31], we determined whether STING is also activated by the TOP1 inhibitors. Firstly, we used IF microscopy to detect STING in treated *vs*. untreated cells. The results showed that STING is localised in the perinuclear region of CPT- and LMP776-treated HeLa cells (Fig. 3c). The IF signal is therefore consistent with the mobilisation of activated STING to the Golgi apparatus [32]. As we noticed a strong IF signal after drug treatments (Fig. 3c), we then determined STING transcript and protein levels after drug treatments. TOP1 poisons increased of STING mRNA and protein levels whereas cGAS protein and mRNA levels were not substantially increased (Fig. 3d and Supplementary Fig. S6). These data indicate that TOP1 poison may also lead to an upregulation of STING and cGAS.

As STING leads to the activation of inducible transcription factors (mainly IRF3 and NF-kB) and IRF3- and type I IFN-dependent genes, we determined the expression levels of immune genes in HeLa cells treated with CPT or LMP776, and their dependence on STING activity. In particular, we evaluated a number of immune genes that can be split into two general categories: first, IRF3-dependent or IFN-β-stimulated genes (CCL5, CXCL10, DDX60, IFI44, IFIT1, ISG15, IFNB1 and IFNA1), which can also be activated by NF-kB; second, immune genes dependent mainly on NF-kB (CCL20, CXCL1, IL1A, IL1B, IL6, IL8 and TNF) (Fig. 3e). We found that TOP1 poisons induced the expression of almost all the studied genes (with the exception of IFNA1) within 48 h from drug removal in HeLa cells (Fig. 3e). Although IRF3-dependent CCL5 and CXCL10 genes were the most induced genes, the induction rate was generally in the same range for the two categories (TNF was the least induced gene), suggesting that Top1 poisons, under our conditions, activated either NF-kB- and IRF3-inducible genes (Fig. 3e). LMP776 was slightly more active than CPT, consistently with STING and cGAS activation (Fig. 3b–d and Supplementary Fig. S6).

**Fig. 4:**
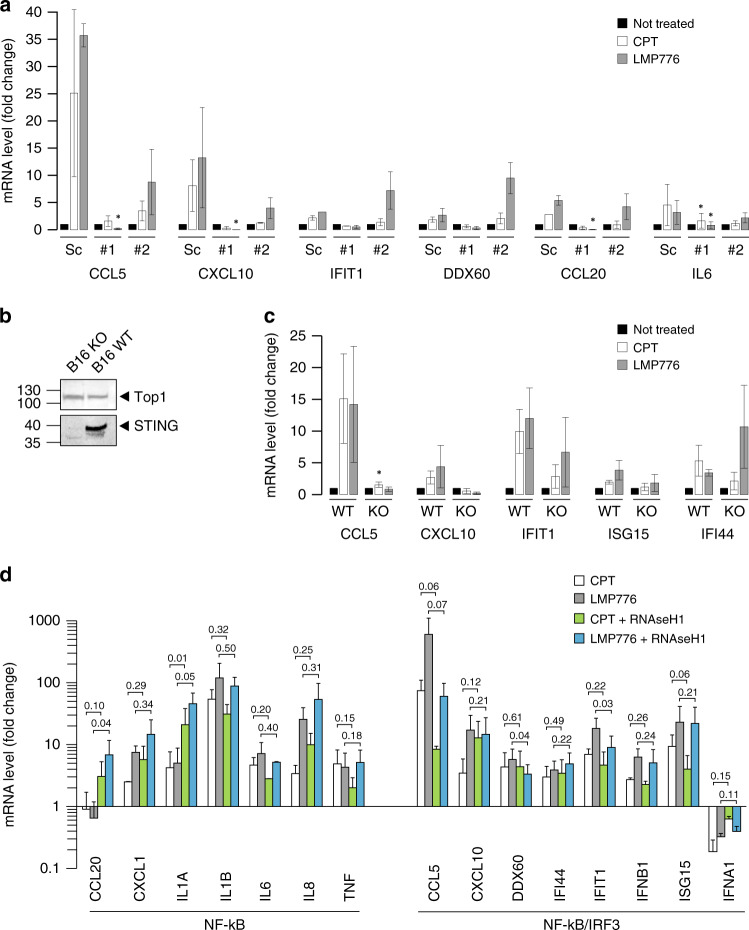
cGAS/STING pathway-dependent cytokine expression in HeLa cells. **a** Cytokines expression in HeLa cells transfected with scrambled siRNA (Sc) or two different siRNA against STING (#1 and #2). Asterisks indicate statistical significance of silenced sample compared to scramble by *t* test. *P < 0.05. **b** WB of STING protein level in not treated B16 WT and KO for STING cell line. **c** Cytokines expression was analysed as in panel **a** in both WT and KO for STING B16 cell. Asterisks indicate statistical significance in comparison with untreated cells by *t* test. *P < 0.05, **P < 0.01. **d** Expression level of cytokine genes in HeLa overexpressing RNaseH1 cell line in response to CPT and LMP776. Data are represented as mean of two biological replicates ± SEM; *P* values are indicated on the top of each bar and they are calculated as one-tailed ratio paired *t* test.

Next, we determined gene expression levels in HeLa cells following STING silencing with two different siRNAs that decreased or abolished STING gene transcripts and/or protein levels (Supplementary Fig. S7A, B). The results showed that STING gene silencing strongly reduced transcription activation of both NF-kB- and IRF3-inducible genes (Fig. 4a) demonstrating that TOP1 poison-induced immune gene activation is mainly due to the STING signalling pathway. To further support the role of STING in TOP1 poison-induced immune gene expression, we determined immune gene activation by LMP776 and CPT in a different cell model, the murine melanoma B16 cell line, by using STING gene wild-type (wt) or CRISPR-KO B16 cell lines (Fig. 4b) [13]. CPT and LMP776 were able to stimulate the transcription of the tested immune genes in wt but not in STING-KO B16 cells (Fig. 4c), showing that the role of STING in immune gene activation by TOP1 poisons is not restricted to human cancer cells.

In addition, we determined the effects of RNaseH1 overexpression on CPT and LMP776 activation of immune genes (Fig. 4d). RNaseH1 increased the expression of CCL20, CXCL1, IL1A and IL8 genes, whereas it reduced the expression of CCL5, DDX60, IFIT1and ISG15 (Fig. 4d), leaving unaltered other genes. RNaseH1 effects are only partial as delayed drug effects are likely mediated by direct and indirect mechanisms. Thus, Top1 poison induction of IRF3-dependent genes, but not NF-kB-dependent genes, is partially dependent on R-loops, in agreement with the R-loop role in the induction of genome instability by Top1 poisons.

Taken altogether, the above findings demonstrate that LMP776 and CPT activate the cytoplasmic cGAS/STING signalling pathway and the expression of immune genes in human HeLa and murine B16 cancer cells in a manner dependent on STING activation.

### TOP1 poison-induced immune gene activation is impaired in human SCLC cell lines

**Fig. 5:**
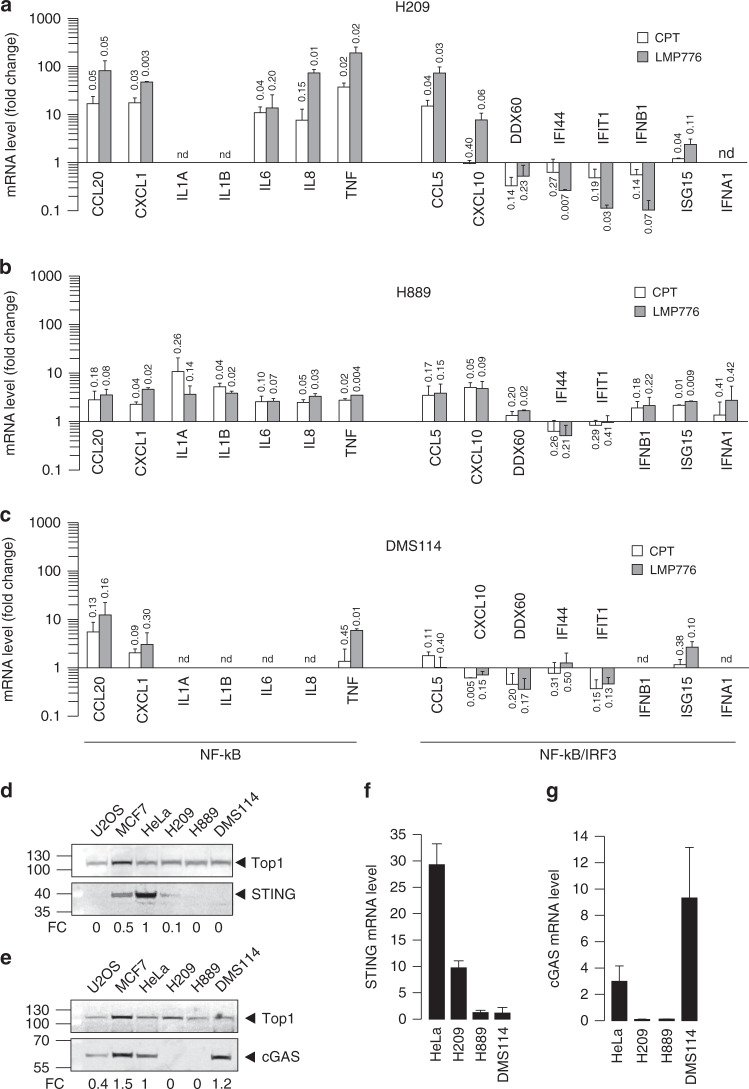
Cytokines expression levels in SCLC. Expression level of a panel of cytokine genes in (**a**) H209, (**b**) H889 and (**c**) DMS114 cell line in response to CPT and LMP776. Data are represented as mean of two biological replicates ± SEM; *P* values are indicated on the top of each bar and they are calculated as one-tailed ratio paired *t* test; same experimental condition of Fig. 1a. For (**a**–**c**), nd = not determined value because under detection level. Western blot of (**d**) STING protein and (**e**) cGAS protein level in several cell types. TOP1 protein levels are also shown. Red ponceau as loading control is in Supplementary Fig. S6. Fold change (FC) represent quantification values compared to HeLa cells (normalised on red ponceau). mRNA quantification of (**f**) STING and (**g**) cGAS gene level in HeLa and lung cancer cell lines. Expression levels were determined by qRT-PCR and normalised on cytochrome *c* transcript.

As LMP776 and CPT induced similar micronuclei levels in HeLa and SCLC cancer cell lines (Fig. 1), we next tested whether the studied TOP1 poisons could also activate immune gene expression in H209, H889 and DMS114 cells (human SCLC lines). Overall, gene activation was lower in SCLC than HeLa cells for both LMP776 and CPT, even if the former was slightly more effective than the latter (Fig. 5a–c). The tested SCLC cell lines responded differently to the TOP1 poisons as NF-kB-dependent genes were somewhat more activated than IRF3-dependent genes in H209 cells, whereas induction of all immune genes was reduced in H889 and DMS114 cells (Fig. 5b, c). As these results were in contrast with micronuclei levels induced by the drugs, we wondered whether the cGAS/STING pathway was functional, and then determined the expression levels of STING and cGAS in the three SCLC cell lines. The results show that STING was expressed in H209, but not in H889 and DMS114 cells (Fig. 5d, f), whereas cGAS was expressed in DMS114, but not in H209 and H889 cells (Fig. 5e, g). STING protein levels were always lower in SCLC than HeLa and MCF7 cells (Fig. 5d, f), suggesting that the low activation of immune genes in H209 cells could be due to reduced STING expression in H209 cells. Similarly to HeLa cells, chemical inhibition of STING activity with H151 [33] in H209 effectively reduced gene activation by Top1 poisons (Supplementary Fig. S8). In addition, as cGAS is not detectable in H209 and the induced gene signature is related to NF-kB, we cannot exclude that a non-canonical STING activation is operative in H209 [34]. The lack of gene activation in H889 and DMS114 cells could be due to undetectable STING levels in those cells (Fig. 5d, f).

As methylation of STING gene can result in a reduced expression in SCLC cells (see below), we treated DMS114 cells with 5’-azacytidine (a demethylating agent, [35]) to increase STING expression (Supplementary Fig. S9A on the right), however, Top1 poisons were still not able to activate immune genes (Supplementary Fig. S9A). We then overexpressed STING exogenously (Supplementary Fig. S9B on the right), however, even with a high cellular content of STING, Top1 poisons could not effectively activate immune genes (Supplementary Fig. S9B).

Thus, altogether our findings show that immune gene activation induced by LMP776 and CPT is markedly impaired in SCLC cells likely due to a defective STING pathway.

### Altered expression of STING pathway genes in human SCLC tumours associated with tumour immunological cold features

As the findings pointed to the expression of STING and cGAS for immune gene activation in cancer cells, we next turn to human lung tumour datasets to analyse expression levels of STING pathway genes in relation to immunological tumour features.

First, we analysed CNV and mutations of STING, cGAS and other STING pathway genes in human lung cancers and 29 other cancer types across ~7800 tumour samples from The Cancer Genome Atlas (TCGA) [36]. Our analysis of mutation rates showed that the four STING pathway genes (cGAS, STING, TBK1 and IRF3) were usually not mutated in human cancers, with a complete absence of homozygous loss-of-function mutations in almost all cancer types (Supplementary Fig. S10A). Most of the observed low-frequency mutations are weak gene amplification and heterozygous loss-of-function mutations (Supplementary Fig. S10A). We further investigated the interplay of the cGAS/STING pathway and tumour immune microenvironment, computing correlations of gene expression with leucocyte fraction and other scores regarding signatures of immune cell infiltration in tumour samples and with gene set enrichment scores regarding the induction of innate immune response [37]. This analysis showed that STING gene expression is markedly positively correlated with immune cells infiltration and interferon response in almost all cancer types, underlining its role in innate immune response activation in cancer (Supplementary Fig. S10B). cGAS gene expression is well correlated in many cancer types (Supplementary Fig. S10B), while TBK1 and IRF3 gene expression is correlated with immune cells presence and interferon response only in few specific cancer types (Supplementary Fig. S10B).

Next, we focused on gene expression variations of these genes in four human lung cancer subtypes. We used RNA-seq data of two non-small-cell lung cancer (NSCLC) types, lung adenocarcinoma (LUAD, *n* = 510 tumour samples and 55 normal samples) and lung squamous cell carcinoma (LUSC, *n* = 496 tumour samples and 47 normal samples) from publicly available TCGA database. In addition, we analysed a SCLC (*n* = 74) and large-cell neuroendocrine lung cancer (LCNEC, *n* = 40) datasets from the European Genome-Phenome Archive (EGA, EMBL-EBI) [1, 38]. Raw gene counts for each cancer types were collected, batch corrected and normalised for further analysis (Supplementary Fig. S11A).

**Fig. 6:**
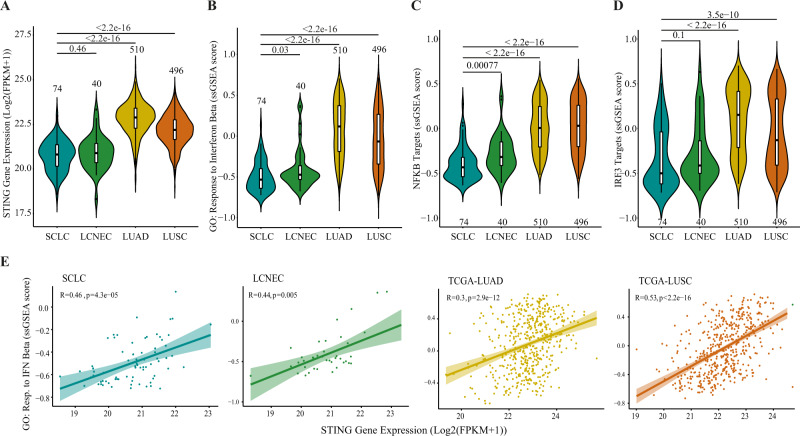
STING gene expression is downregulated in SCLC and correlates with interferon response activation. **a** Violin plot of STING gene expression across different lung cancer histopathology types. *P* value of the Wilcoxon test and the number of samples for each group are reported in the plot. **b** Violin plot of ssGSEA enrichment score of “GO: Response to Interferon Beta” gene set across different lung cancer histopathology types. *P* value of Wilcoxon test and the number of samples for each group are reported in the plot. **c**, **d** Violin plot of ssGSEA enrichment score of NF-kB-target and IRF-target gene sets, respectively, across different lung cancer histopathology types. *P* value of the Wilcoxon test and the number of samples for each group are reported in the plot. **e** Scatter plots of STING gene expression correlated to ssGSEA enrichment score of “GO: Response to Interferon Beta” gene set for each lung cancer type. Spearman correlation coefficient (R) and *P* value of correlation test are reported in each graph.

We analysed four genes of the cGAS/STING pathway (cGAS, STING, TBK1 and IRF3). and the results showed that STING is significantly downregulated in SCLC and LCNEC compared to LUAD and LUSC types (Fig. 6a). cGAS expression showed no expression difference among lung cancer types (Supplementary Fig. S11B), while TBK1 and IRF3 showed a low downregulation only in SCLC tumour samples (Supplementary Fig. S11C, D). Notably, the expression of NF-kB subunits is significantly downregulated similarly to IRF3 expression (Supplementary Fig. S11E). Comparing gene expression of tumours to matched normal tissues, available only for TCGA LUAD and LUSC cancers [39, 40], we observed a slight upregulation, on average, of IRF3 in both LUAD and LUSC (*P*val = 7.111e-07 and 4.117e-06, respectively). Interestingly, we observed a strong downregulation of STING gene expression in both LUAD (*P*val = 1.626e-07) and LUSC (*P*val = 3.637e-12) (Supplementary Fig. S11F, G), suggesting that STING downregulation in SCLC and LCNEC types is even stronger in comparison to normal lung tissues. cGAS was found slightly upregulated only in LUAD tumour samples compared to normal tissues (2.511e-06) while TBK1 showed no significant expression changes between tumour and normal samples (Supplementary Fig. S11F, G). Thus, these data show that STING is specifically downregulated in SCLC and LCNEC tumours in comparison to other lung cancer types and, likely, to normal lung tissues.

We further investigated the mechanism of STING downregulation using a publicly available methylation dataset for LUAD and LUSC cohorts. We observed that STING gene is subjected to a significant hypermethylation in both LUAD (*n* = 17, *P*val = 0.0177) and LUSC (*n* = 7, *P*val = 0.0200) in tumour samples compared to matched normal samples (Supplementary Fig. S11H). Furthermore, through a survey of Cancer Cell Line Encyclopaedia (CCLE) data [41], we observed that small-cell lung cancer cell lines DMS114, H889, and, to a lower extent, H209, show a high methylation level of STING gene that strongly affects gene expression, that is lower than STING expression in HeLa cells (Supplementary Fig. S11I).

To assess the status of innate immune activity and its relation to the cGAS/STING pathway key genes in lung cancer types, we then performed a Gene Set Variation Analysis (GSVA) for GO process “*Response to Interferon Beta”* gene set. The results showed that there is a strong negative enrichment for this gene set in LCNEC and, particularly, SCLC samples (Fig. 6b). In order to test whether in SCLC a NF-kB-specific response may be exerted, we performed the GSVA analysis also using two gene sets related to NF-kB and IRF targets derived from TRANSFAC database [17]. We found that both signatures are significantly downregulated in SCLC tumours (Fig. 6c, d). We further confirm these findings by looking at specific NF-kB-driven genes (Supplementary Fig. S12A) and IRF3-dependent genes (Supplementary Fig. S12B) showing an overall downregulation of all the tested genes in SCLC, which is more significative for NF-kB-dependent genes (Supplementary Fig. S12A).

As *STING* expression was reduced in SCLC subtype as interferon response, we performed a correlation analysis of *STING* expression and “*Response to Interferon Beta”* gene set enrichment for each cancer type showing a positive but slight correlation between *STING* and “*Response to Interferon Beta”* GO process in LUSC and neuroendocrine cancer types (SCLC and LCNEC) but not in LUAD (Fig. 6e).

Moreover, investigating CellMiner Cross Database (CellMinerCDB) data [42], we observed that STING expression in lung cancer cell lines is positively correlated (*P*val = 4.6e-16) with antigen-presenting machinery score (Supplementary Fig. S12C), a prediction index for tumour response to immune checkpoint inhibitors [43], while negatively correlates (*P*val = 5.4e-30) with STING gene methylation (Supplementary Fig. S12D), consistently with observation in TCGA LUAD and LUSC samples.

Overall, our findings indicate that STING expression is generally downregulated in human lung cancers compared to normal tissues, and that its expression is more downregulated in SCLC and LCNEC, in agreement with experimental data on SCLC cell lines. In SCLC cell lines and LUAD and LUSC cohorts, the downregulation is associated with STING promoter hypermethylation. STING downregulation in SCLC tumours also correlates with a lower expression of cGAS/STING pathway-dependent genes, for both NF-kB- and IRF3-related responses.

## Discussion

Our findings show that anticancer TOP1 poisons, CPT and LMP776, increase micronuclei generation with a mechanism involving R-loop accumulation, leading to activation of the cGAS-STING pathway and immune gene expression in HeLa cancer cells. In addition, we show that Top1 poisons can activate both IRF3- and NF-kB-dependent genes, however, they cannot in SCLC cancer cells as the cGAS-STING pathway is markedly impaired likely due to several mechanisms including a drastic reduction of STING and/or cGAS expression.

In agreement with a previous report on G-quadruplex binders and micronuclei in mammalian cancer cells [16, 19], abolishing TOP1 poison-mediated increase of R-loops by RNaseH1 overexpression drastically reduced micronuclei levels (Fig. 1). As TOP1 poisons inhibit replication fork progression leading to replication stress and DSBs through an increase of nuclear R-loops (Fig. 2 and ref. [22]), the findings overall support a role for R-loops and replication stress in micronuclei production at mitosis [10]. Top1 poison-induced micronuclei can activate immune genes via the classical cGAS/STING pathway, as shown by results in HeLa cells. However, our data cannot exclude a cGAS-independent activation of STING pathway by Top1 poisons leading to immune gene activation via NF-kB [34] (Fig. 3). Our results show that *STING* and *cGAS* expression is often markedly reduced or abolished in SCLC cell lines and tumours, thus impairing the activity of the STING pathway and the activation of immune genes (Figs. 4–6). Accordingly, human SCLC cells are generally resistant to the modulation of innate immune genes by DNA-damaging agents through cytoplasmic pattern recognition pathways. Interestingly, in three different SCLC cell lines (H82, H526 and H1048) wherein STING and cGAS genes are expressed, PARP inhibitors were shown to increase the cellular levels of IFN-B gene mRNA along with activated pathway factors such as pSTING_S366 and cGAS [44]. Our data are also in agreement with evidence that low CPT doses can activate the cGAS-STING pathway in a different cell model [29]. Thus, overall, the data document that the expression of STING can be critical for the signalling pathway functionality in SCLC cells. As STING expression is often reduced due to gene promoter hypermethylation in human lung cancer cells (Supplementary Fig. S11I) and patient tumour samples (Supplementary Fig. S11H) [45], one might assume that demethylating agents could be useful to restore STING expression and pathway activity in SCLC. In contrast, our data demonstrate that immune gene expression was not activated by Top1 poisons even in the presence of 5’-azacytidine and in STING-overexpressed cells, suggesting that additional players are involved in the impairment of cGAS/STING pathways in SCLC.

As chemotherapy, including TOP1 poisons, remains the most effective treatment of SCLC patients, understanding the mechanisms triggered by anticancer TOP1 poisons can open novel opportunities to improve SCLC treatments. An important aspect of our findings is that sub-cytotoxic concentrations of CPT and LMP776 trigger micronuclei production and innate immune gene activation at later times (48 h) following cell recovery in a drug-free medium. Therefore, TOP1 poisons can likely be endowed with clinically-effective antitumor activity due to both a cell-killing action at high concentrations as well as immunomodulatory effects at lower, less cytotoxic concentrations, suggesting that appropriate doses could be used to achieve immunostimulatory effects in patients [46]. A previous report documented that CPT and topotecan instead reduced IFN gene and ISG expression showing antiviral activity against influenza A virus and Sendai virus strains [47]. In this case, however, TOP1 poisons were used at cytotoxic concentrations for short times (4–16 h) and the effects on gene transcription were correlated to effective inhibition of RNA polymerase II by TOP1 poisons [3, 4].

Interestingly, TOP1 poisons at relatively low doses have been shown to elicit other delayed effects relevant to immunological responses in several cancer models. Topotecan was shown to upregulate the expression of MHC class I through elevated expression of IFN-β and activation of type I IFN signalling after 4-day treatments of breast cancer cells [48]. In addition, TOP1 poisons have been shown to enhance recognition of patient melanoma cells by T cells and T cell-mediated cytotoxicity with a different mechanism involving p53 activity [49]. In a syngeneic triple-negative breast cancer mouse model, topotecan was shown to activate STING and the release of DNA-containing exosomes which trigger the activation of dendritic cells and CD8 + T cells. Importantly, the antitumor activity was decreased in mice lacking STING [50].

Published results show that different cancers upregulate cGAS/STING pathway, likely causing chronic inflammation [51] leading to the survival of tumour cells, cancer progression and high resistance towards cytotoxic agents [52, 53]. In addition, a prevalent stimulation of NF-kB- rather than IRF3-signature after STING activation, is associated with the senescence-associated secretory phenotype (SASP), which is associated with chronic inflammation and tissue destruction [54]. Therefore, immunomodulatory effects of Top1 poisons likely depend on patient-specific molecular features of human cancers. Overall, our data show that, depending on cancer type, TOP1 poisons have immunostimulatory effects mainly due to the STING/cGAS signalling pathway, although potential immunotherapy protocols should be carefully evaluated as SCLC may downregulate the pathway abolishing the drug-triggered immunomodulating activity.

## Supplementary information

Supplementary Information

## Data Availability

Gene expression data and clinical data of human small-cell lung cancer samples are deposited at EMBL-EGA Data Archive under accession numbers EGAD00001001244, EGAD00001003801 and EGAD00001001431. TCGA data for human lung adenocarcinoma (LUAD) and lung squamous cell carcinoma (LUSC) are stored on UCSC Xena Browser datasets page (https://xenabrowser.net/datapages/).

## References

[1] George J, Lim JS, Jang SJ, Cun Y, Ozretia L, Kong G (2015). Comprehensive genomic profiles of small cell lung cancer. Nature..

[2] Thomas A, Pommier Y (2016). Small cell lung cancer: time to revisit DNA-damaging chemotherapy. Sci Transl Med.

[3] Pommier Y (2006). Topoisomerase I inhibitors: camptothecins and beyond. Nat Rev Cancer.

[4] Capranico G, Marinello J, Chillemi G (2017). Type I DNA topoisomerases. J Med Chem.

[5] Pommier Y, Sun Y, Huang SYN, Nitiss JL (2016). Roles of eukaryotic topoisomerases in transcription, replication and genomic stability. Nat Rev Mol Cell Biol.

[6] Thomas A, Pommier Y (2019). Targeting topoisomerase I in the era of precision medicine. Clin Cancer Res.

[7] García-Muse T, Aguilera A (2016). Transcription-replication conflicts: how they occur and how they are resolved. Nat Rev Mol Cell Biol.

[8] Hamperl S, Bocek MJ, Saldivar JC, Swigut T, Cimprich KA (2017). Transcription-replication conflict orientation modulates R-loop levels and activates distinct DNA damage responses. Cell..

[9] Chan KL, Palmai-Pallag T, Ying S, Hickson ID (2009). Replication stress induces sister-chromatid bridging at fragile site loci in mitosis. Nat Cell Biol.

[10] Liu Y, Nielsen CF, Yao Q, Hickson ID (2014). The origins and processing of ultra fine anaphase DNA bridges. Curr Opin Genet Dev.

[11] Holmström M, Winters V (1992). Micronucleus induction by camptothecin and amsacrine in bone marrow of male and female CD-1 mice. Mutagenesis..

[12] MacKenzie KJ, Carroll P, Martin CA, Murina O, Fluteau A, Simpson DJ (2017). CGAS surveillance of micronuclei links genome instability to innate immunity. Nature..

[13] Harding SM, Benci JL, Irianto J, Discher DE, Minn AJ, Greenberg RA (2017). Mitotic progression following DNA damage enables pattern recognition within micronuclei. Nature..

[14] Thomas A, Takahashi N, Rajapakse VN, Zhang X, Sun Y, Ceribelli M (2021). Therapeutic targeting of ATR yields durable regressions in small cell lung cancers with high replication stress. Cancer Cell.

[15] Gotwals P, Cameron S, Cipolletta D, Cremasco V, Crystal A, Hewes B (2017). Prospects for combining targeted and conventional cancer therapy with immunotherapy. Nat Rev Cancer.

[16] Miglietta G, Russo M, Capranico G (2020). G-quadruplex–R-loop interactions and the mechanism of anticancer G-quadruplex binders. Nucleic Acids Res.

[17] Miglietta G, Russo M, Duardo RC, Capranico G (2021). G-quadruplex binders as cytostatic modulators of innate immune genes in cancer cells. Nucleic Acids Res.

[18] Tlemsani C, Pongor L, Elloumi F, Girard L, Huffman KE, Roper N (2020). SCLC-CellMiner: a resource for small cell lung cancer cell line genomics and pharmacology based on genomic signatures. Cell Rep..

[19] De Magis A, Manzo SG, Russo M, Marinello J, Morigi R, Sordet O (2019). DNA damage and genome instability by G-quadruplex ligands are mediated by R loops in human cancer cells. Proc Natl Acad Sci USA.

[20] Repana D, Nulsen J, Dressler L, Bortolomeazzi M, Venkata SK, Tourna A (2019). The Network of Cancer Genes (NCG): a comprehensive catalogue of known and candidate cancer genes from cancer sequencing screens. Genome Biol.

[21] Ohle C, Tesorero R, Schermann G, Dobrev N, Sinning I, Fischer T (2016). Transient RNA-DNA hybrids are required for efficient double-strand break repair. Cell..

[22] Marinello J, Chillemi G, Bueno S, Manzo SG, Capranico G (2013). Antisense transcripts enhanced by camptothecin at divergent CpG-island promoters associated with bursts of topoisomerase I-DNA cleavage complex and R-loop formation. Nucleic Acids Res.

[23] Phillips DD, Garboczi DN, Singh K, Hu Z, Leppla SH, Leysath CE (2013). The sub-nanomolar binding of DNA-RNA hybrids by the single-chain Fv fragment of antibody S9.6. J Mol Recognit..

[24] Antony S, Agama KK, Miao ZH, Takagi K, Wright MH, Robles AI (2007). Novel indenoisoquinolines NSC 725776 and NSC 724998 produce persistent topoisomerase I cleavage complexes and overcome multidrug resistance. Cancer Res.

[25] Aguilera A, Gómez-González B (2017). DNA-RNA hybrids: the risks of DNA breakage during transcription. Nat Struct Mol Biol.

[26] Bou-Nader C, Bothra A, Garboczi DN, Leppla SH, Zhang J (2022). Structural basis of R-loop recognition by the S9.6 monoclonal antibody. Nat Commun..

[27] Cristini A, Ricci G, Britton S, Salimbeni S, Huang SYN, Marinello J (2019). Dual processing of R-loops and topoisomerase I induces transcription-dependent DNA double-strand breaks. Cell Rep..

[28] Cristini A, Géraud M, Sordet O. Transcription-associated DNA breaks and cancer: a matter of DNA topology. Int Rev Cell Mol Biol. 2021;364:195–240. 10.1016/bs.ircmb.2021.05.001 34507784

[29] Pépin G, Nejad C, Ferrand J, Thomas BJ, Stunden HJ, Sanij E, et al. Topoisomerase 1 inhibition promotes cyclic GMP-AMP synthase-dependent antiviral responses. MBio. 2017;8:e01611-17. 10.1128/mBio.01611-17 PMC5626974 28974621

[30] Al-Asmari SS, Rajapakse A, Ullah TR, Pépin G, Croft LV, Gantier MP (2022). Pharmacological targeting of STING-dependent IL-6 production in cancer cells. Front Cell Dev Biol.

[31] Wu J, Sun L, Chen X, Du F, Shi H, Chen C (2013). Cyclic GMP-AMP is an endogenous second messenger in innate immune signaling by cytosolic DNA. Science..

[32] Mukai K, Konno H, Akiba T, Uemura T, Waguri S, Kobayashi T (2016). Activation of STING requires palmitoylation at the Golgi. Nat Commun.

[33] Haag SM, Gulen MF, Reymond L, Gibelin A, Abrami L, Decout A (2018). Targeting STING with covalent small-molecule inhibitors. Nature..

[34] Dunphy G, Flannery SM, Almine JF, Connolly DJ, Paulus C, Jønsson KL (2018). Non-canonical activation of the DNA sensing adaptor STING by ATM and IFI16 mediates NF-κB signaling after nuclear DNA damage. Mol Cell.

[35] Tsuji-Takayama K, Inoue T, Ijiri Y, Otani T, Motoda R, Nakamura S (2004). Demethylating agent, 5-azacytidine, reverses differentiation of embryonic stem cells. Biochem Biophys Res Commun.

[36] Hoadley KA, Yau C, Hinoue T, Wolf DM, Lazar AJ, Drill E (2018). Cell-of-origin patterns dominate the molecular classification of 10,000 tumors from 33 types of cancer. Cell..

[37] Thorsson V, Gibbs DL, Brown SD, Wolf D, Bortone DS, Ou Yang TH (2018). The immune landscape of cancer. Immunity..

[38] George J, Walter V, Peifer M, Alexandrov LB, Seidel D, Leenders F (2018). Integrative genomic profiling of large-cell neuroendocrine carcinomas reveals distinct subtypes of high-grade neuroendocrine lung tumors. Nat Commun.

[39] Collisson EA, Campbell JD, Brooks AN, Berger AH, Lee W, Chmielecki J (2014). Comprehensive molecular profiling of lung adenocarcinoma: the cancer genome atlas research network. Nature..

[40] Hammerman PS, Voet D, Lawrence MS, Voet D, Jing R, Cibulskis K (2012). Comprehensive genomic characterization of squamous cell lung cancers. Nature..

[41] Ghandi M, Huang FW, Jané-Valbuena J, Kryukov GV, Lo CC, McDonald ER (2019). Next-generation characterization of the Cancer Cell Line Encyclopedia. Nature..

[42] Luna A, Elloumi F, Varma S, Wang Y, Rajapakse VN, Aladjem MI (2021). CellMiner cross-database (CellMinerCDB) version 1.2: exploration of patient-derived cancer cell line pharmacogenomics. Nucleic Acids Res.

[43] Wang S, He Z, Wang X, Li H, Liu XS. Antigen presentation and tumor immunogenicity in cancer immunotherapy response prediction. eLife. 2019;8:e49020. 10.7554/eLife.49020 PMC6879305 31767055

[44] Sen T, Rodriguez BL, Chen L, Della Corte CM, Morikawa N, Fujimoto J (2019). Targeting DNA damage response promotes antitumor immunity through STING-mediated T-cell activation in small cell lung cancer. Cancer Discov.

[45] Poirier JT, Gardner EE, Connis N, Moreira AL, De Stanchina E, Hann CL (2015). DNA methylation in small cell lung cancer defines distinct disease subtypes and correlates with high expression of EZH2. Oncogene..

[46] Galluzzi L, Humeau J, Buqué A, Zitvogel L, Kroemer G (2020). Immunostimulation with chemotherapy in the era of immune checkpoint inhibitors. Nat Rev Clin Oncol.

[47] Rialdi A, Campisi L, Zhao N, Lagda AC, Pietzsch C, Ho JSY (2016). Topoisomerase 1 inhibition suppresses inflammatory genes and protects from death by inflammation. Science..

[48] Wan S, Pestka S, Jubin RG, Lyu YL, Tsai YC, Liu LF (2012). Chemotherapeutics and radiation stimulate MHC class i expression through elevated interferon-beta signaling in breast cancer cells. PLoS ONE.

[49] McKenzie JA, Mbofung RM, Malu S, Zhang M, Ashkin E, Devi S (2018). The effect of topoisomerase I inhibitors on the efficacy of T-cell-based cancer immunotherapy. J Natl Cancer Inst.

[50] Kitai Y, Kawasaki T, Sueyoshi T, Kobiyama K, Ishii KJ, Zou J (2017). DNA-containing exosomes derived from cancer cells treated with topotecan activate a STING-dependent pathway and reinforce antitumor immunity. J Immunol.

[51] Vashi N, Bakhoum SF (2021). The evolution of STING signaling and its involvement in cancer. Trends Biochem Sci.

[52] Cheradame L, Guerrera IC, Gaston J, Schmitt A, Jung V, Goudin N (2021). STING protects breast cancer cells from intrinsic and genotoxic-induced DNA instability via a non-canonical, cell-autonomous pathway. Oncogene..

[53] Ahn J, Xia T, Konno H, Konno K, Ruiz P, Barber GN (2014). Inflammation-driven carcinogenesis is mediated through STING. Nat Commun.

[54] Dou Z, Ghosh K, Vizioli MG, Zhu J, Sen P, Wangensteen KJ (2017). Cytoplasmic chromatin triggers inflammation in senescence and cancer. Nature..

