# Supplementary material for: Topoisomerase I poison-triggered immune gene activation is markedly reduced in human small-cell lung cancers by impairment of the cGAS/STING pathway
Source: Br J Cancer. 2022 Jul 6;127(7):1214–25. doi: 10.1038/s41416-022-01894-4 (PMC9519573; doi:10.1038/s41416-022-01894-4)
Supplement: Supplementary file 1 — Supplementary Information [file 41416_2022_1894_MOESM1_ESM.docx]

**Supplementary file.**

**Topoisomerase I poison-triggered immune gene activation is markedly reduced in human small-cell lung cancers by impairment of cGAS/STING pathway.**

Jessica Marinello, Andrea Arleo, Marco Russo, Maria Delcuratolo, Francesca Ciccarelli,

Yves Pommier and Giovanni Capranico

**Supplementary Figure 1**

**
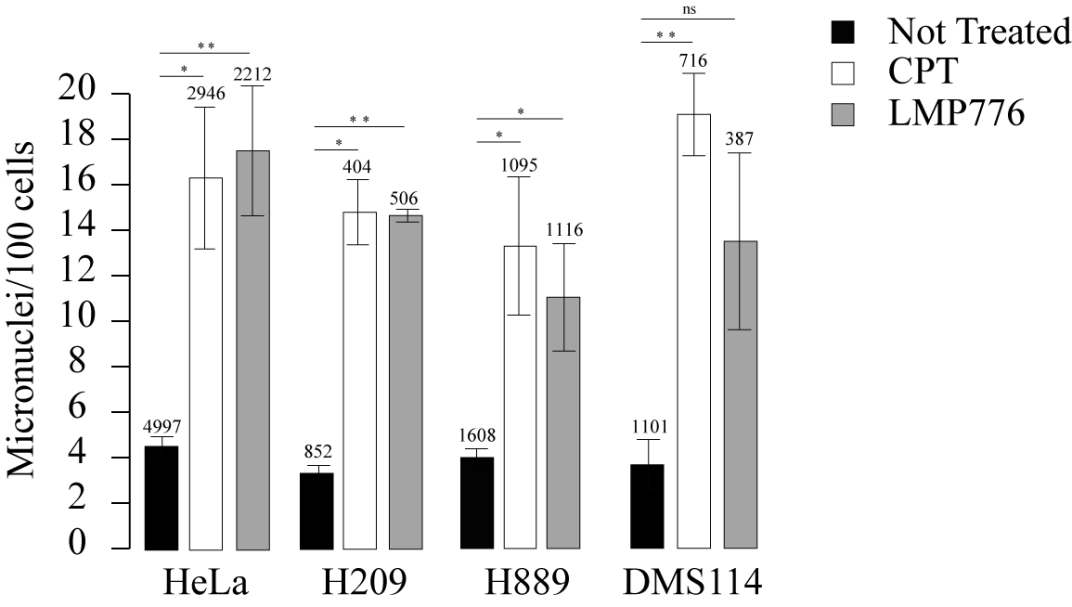
**

**Figure S1.** **Induction of micronuclei by CPT and LMP776 is similar in human HeLa and SCLC H209, H889 and DMS114 cell lines.** Cancer cells were treated for 24 hours with 100 nM CPT or 200 nM LMP776 and then recovered for 48 hours in drug-free medium. Micronuclei were then stained with DAPI and counted with fluorescence imaging. Bars represent the number of micronuclei in 100 cells ± SEM (biological replicates for each cell line are: 5 for HeLa, 2 for H209, 3 for H889, 3 for DMS114). Numbers of analyzed cells are reported above the bar for each sample. Asterisks indicate statistical significance in comparison with untreated cells by t-test. * *p* < 0.05, ** *p* < 0.01.

**Supplementary Figure 2**

**
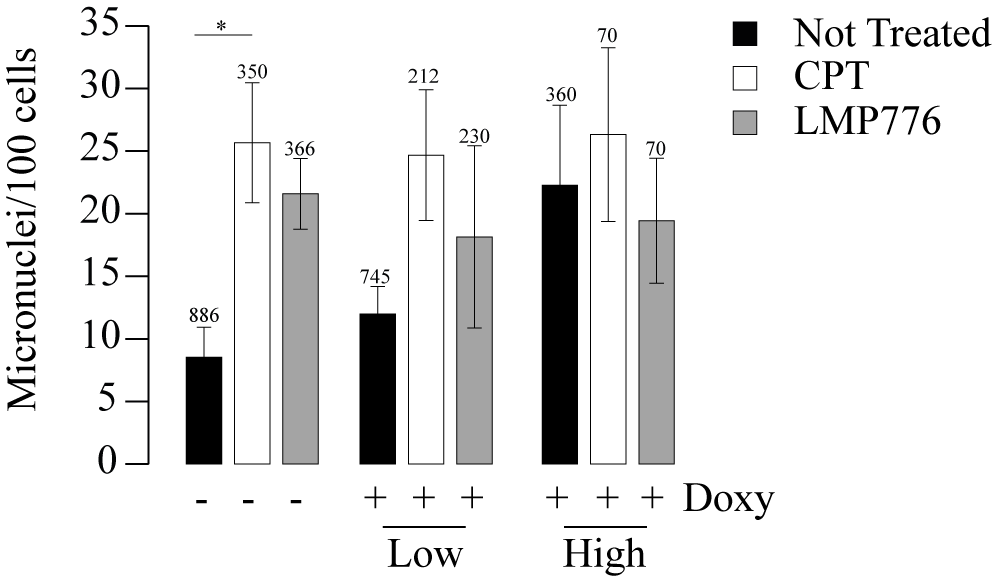
**

**Figure S2**. **Effects of RNAseH1 expression on micronuclei induction by Top1 poisons.**  Micronuclei were counted 48 hours after being released from drug treatment (100 nM CPT or 200 nM LMP776 for 24 hours) in a U2OS_RH cell line, stably transfected with a doxycycline-dependent RNAseH1-expressing vector. U2OS_RH cells were then exposed to doxycicline (+doxy) or not (-doxy) for 48 hours before treatment with Top1 poisons. RNAseH1 expression was detected with an antibody against a FLAG tag fused to the enzyme. RNAseH1-expressing cells were split into two categories: Low and High expression of RNAseH1. Bars represent the number of micronuclei in 100 cells ± SEM (three biological replicates); numbers of analyzed cells are reported above the bar for each sample. Statistical significance is calculated for three independent experiments by t-test: the only significant increase after drug treatment is after CPT-doxy treatment (*p* = 0.019) and borderline for LMP776-doxy (*p* = 0.055). Micronuclei levels are increased by RNAseH1 overexpression (compare Black columns) likely due to functional roles of DNA:RNA hybrids in DSB repair mechanisms [1]. As the number of micronuclei/100 cells is not saturated in untreated and treated cells, CPT and LMP776 have probably lost the ability to increase baseline micronuclei levels when RNAseH1 is overexpressed (High samples).

**Supplementary Figure 3.**


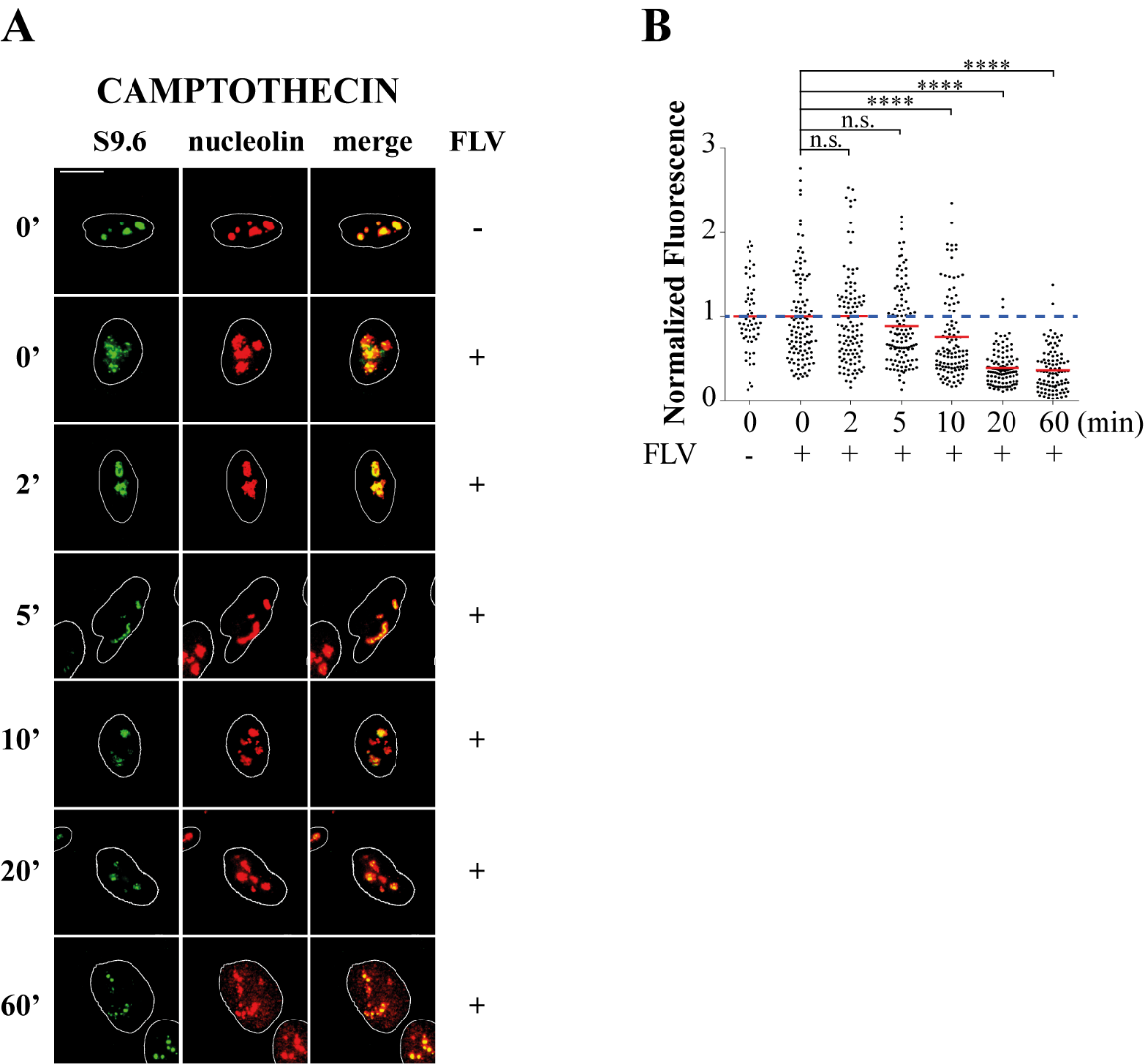


**Figure S3. Specificity of S9.6 fluorescence signals.** Hybrid specificity of S9.6 has recently been demonstrated [2]. At the same time, we assessed hybrid specificity of S9.6 signal under our conditions by using RNAse H1 expression in living cells, as follows: (A) HeLa cells were pretreated or not for 1 hour with Flavopiridol and then Camptothecin was added to the culture medium for the indicated times. R loops nuclear localization was detected by co-staining with S9.6 (green) and anti-nucleolin (red) antibodies. Flavopiridol treatment provokes progressive nucleoli destruction; as a consequence, whole nuclear fluorescence signal was measured. Representative images are reported. Scale bars: 10 μm. Each dot of the quantitation plot in **(B)** represents the normalized fluorescence value of each cell, while red bars show the mean for each sample. Asterisks indicate statistical significance in comparison with untreated cells by the Mann Whitney test. ** p < 0.05, ** p < 0.01, *** p < 0.001, **** p < 0.0001***.** Scale bars: 10 μm.

**Supplementary Figure 4.**


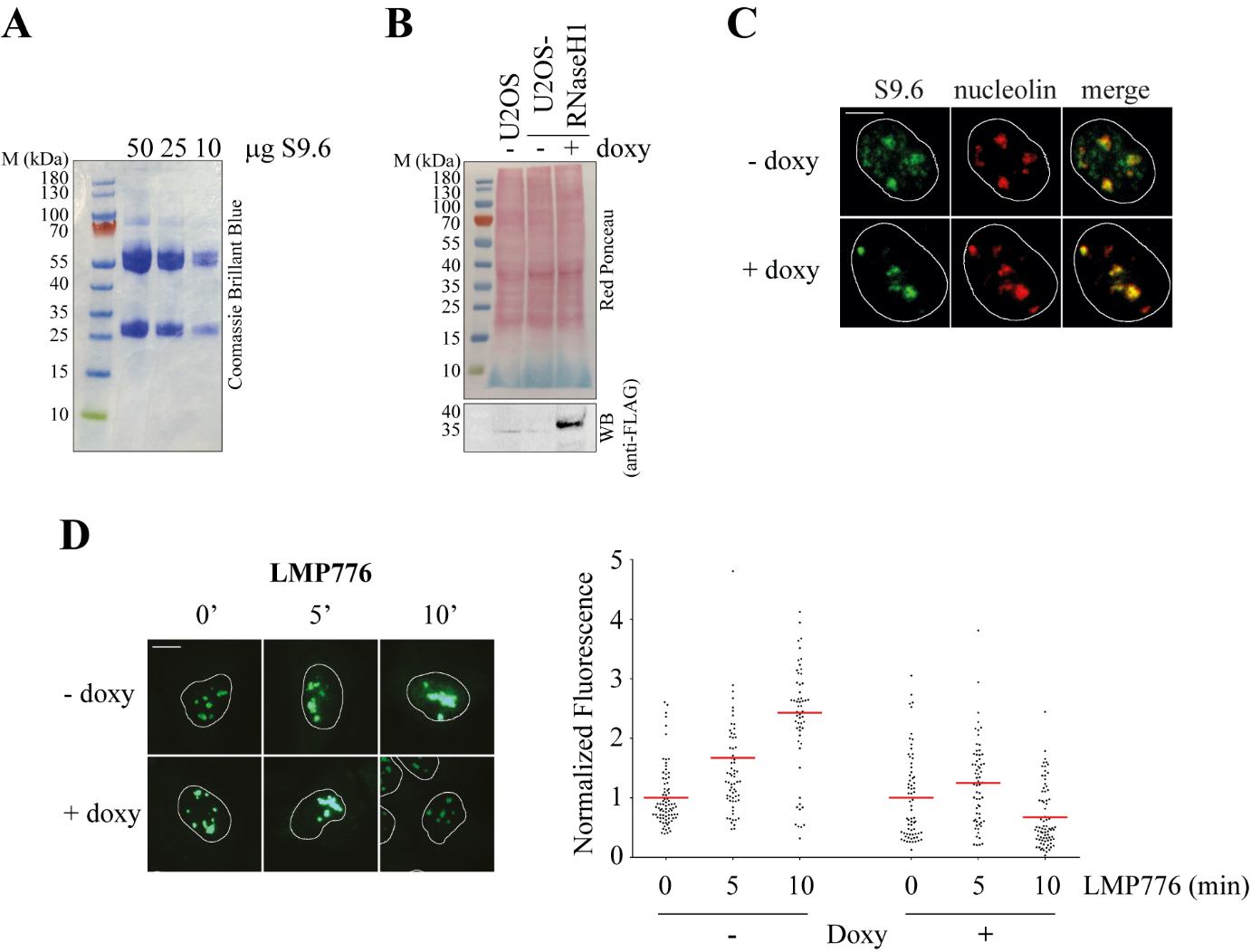


**Figure S4.** **Validation of S9.6. (A)** Comassie Brilliant Blue-stained polyacrylamide gels of S9.6 antibody (different concentration per lane) purified as described in Materials and Methods section. **(B)** WB analysis on total protein extract (red ponceau on top) of U2OS wt and U2OS-RNAseH1 cell line in presence and absence of doxycycline. Antibody against FLAG has been used to detect the expression of exogenous protein in induced cells. **(C)** Effects of exogenous RNAseH1 expression in U2OS-RNAseH1 non-treated cells. Cells were induced or not with doxycycline and then fixed and stained with S9.6 antibody (green) and nucleolin (red). **(D)** Same as in **(C)** but cells were treated for indicated times with LMP776 (10 μM). On the right, quantification of S9.6 signals (as total nuclear fluorescence) is shown. Scale bars: 10 μm.

**Supplementary Figure 5.**

**
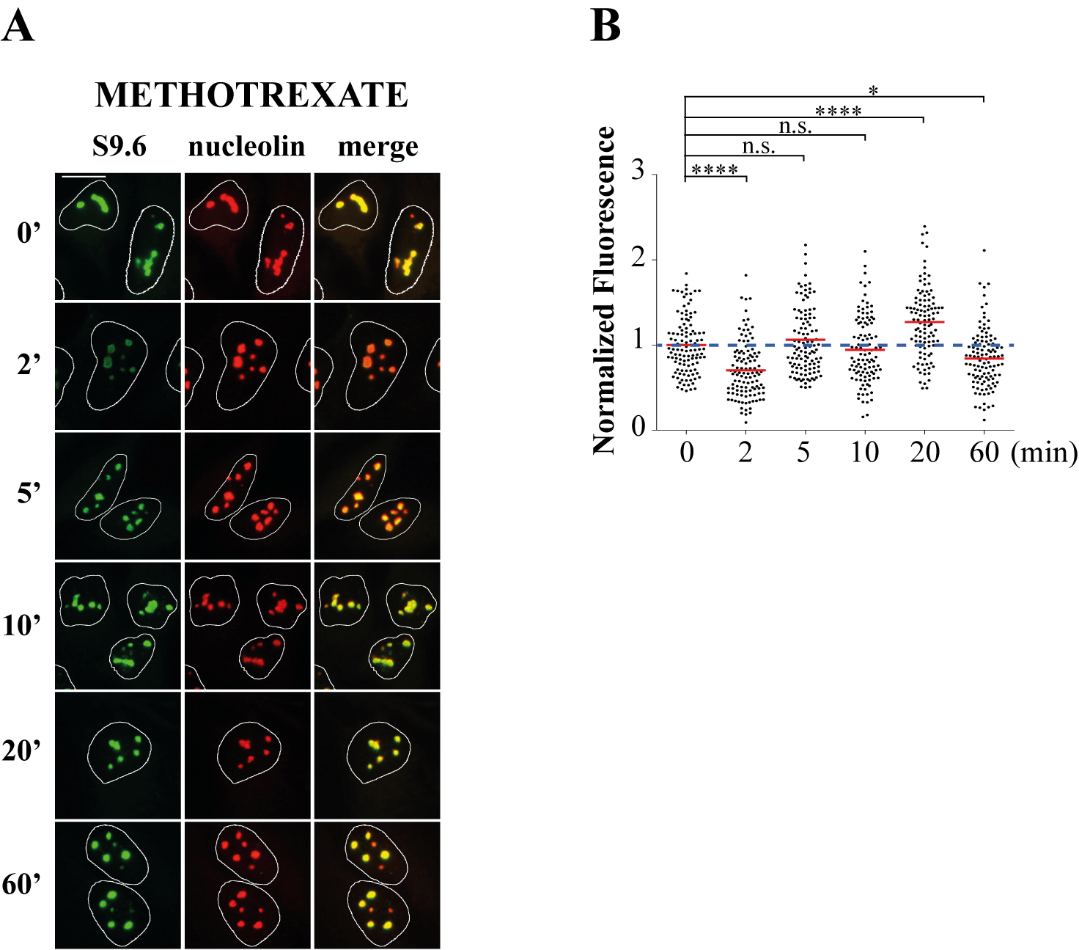
**

**Figure S5.** **Effect of Methotrexate on R loops formation.** **(A)** HeLa cells were treated for indicated times with MTX and R-loops sub-nuclear localization was detected by co-staining with S9.6 (green) and anti-nucleolin (red) antibodies. Representative images are reported; scale bars: 10 μm, Each dot of the quantitation plots in **(B)** represents the normalized fluorescence value of each cell, while red bars show the mean for each sample. Asterisks indicate statistical significance in comparison with untreated cells by the Mann Whitney test. ** p < 0.05, ** p < 0.01, *** p < 0.001, **** p < 0.0001***.** Scale bars: 10 μm.

**Supplementary Figure 6.**


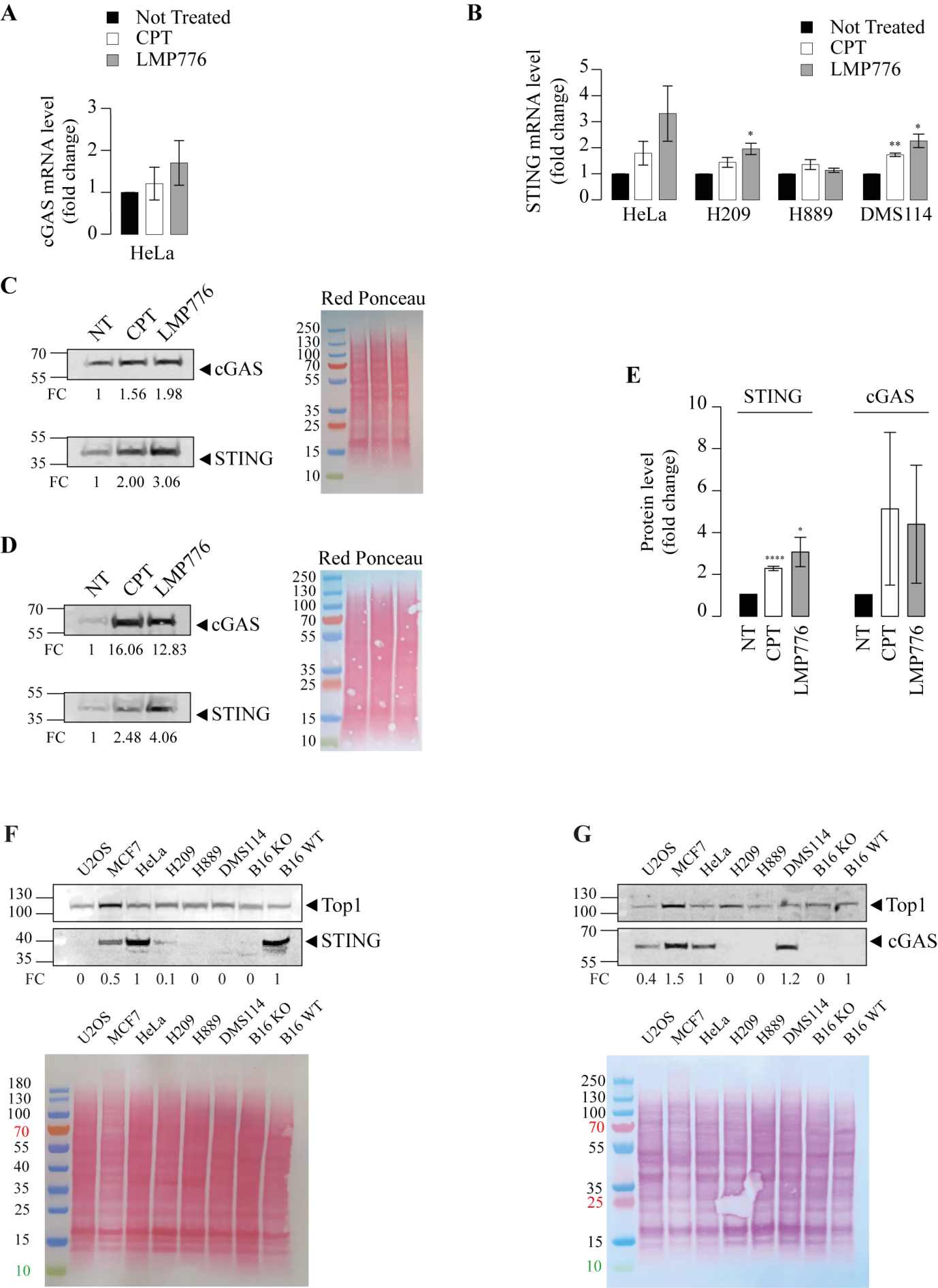


**Figure 6S. Effect of Top1 poisons on cGAS and STING mRNA and proteins level in HeLa cells.** **(A)** cGAS mRNA level was determined by qrt-PCR in non-treated (NT) and treated cells. **(B)** STING mRNA level was determined by qrt-PCR in non-treated (NT) and treated HeLa and lung cancer cells. **(C-D)** Two examples of experimental replicates of cGAS and STING protein WB in non-treated and treated HeLa cells. FC represents fold change in signal intensity of treated cells compared to non-treated cells, normalized on red ponceau (on the right). **(E)** Quantitation of 4 experimental replicates of WB as in C and D. Asterisks indicate statistical significance in comparison with untreated cells by t-test. * *p* < 0.05, ** *p* < 0.01. Western Blot of **(F)** STING protein and **(G)** cGAS protein in several cell types with their respective Red Ponceau images (below). TOP1 is the loading control. Fold change (FC) represent quantification values compared to HeLa cells (normalized on red ponceau) for U2OS, MCF7, HeLa, and lung cancer cells; FC values for B16 cell lines are compared to B16 WT and normalized on red ponceau.

**Supplementary Figure 7.**

**
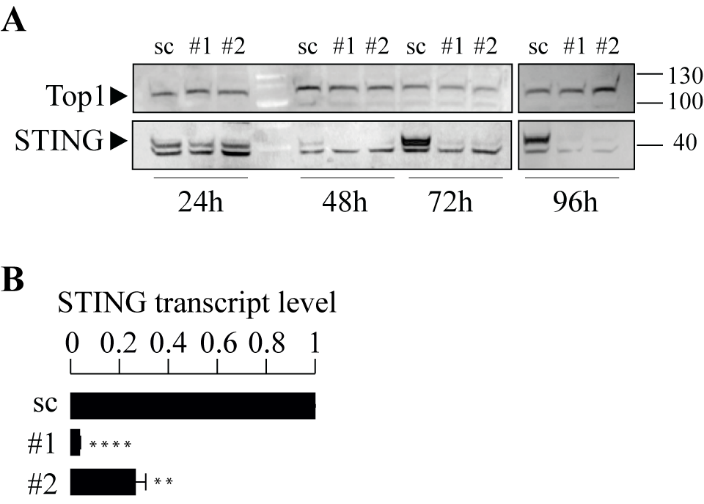
**

**Figure S7. Silencing of STING protein in HeLa cells. (A)** Western Blot of STING protein level in HeLa cells transfected with scrambled siRNA (Sc) or two different siRNA against STING (#1 and #2) at different time points. Top1 is the loading control. **(B)** STING transcript level determined by qrt-PCR in HeLa cells transfected with scrambled siRNA (Sc) or two different siRNA against STING (#1 and #2). Asterisks indicate statistical significance in comparison with scramble transfected cells by t-test. ** p < 0.05, ** p < 0.01, *** p < 0.001, **** p < 0.0001***.**

**Supplementary Figure 8.**

**
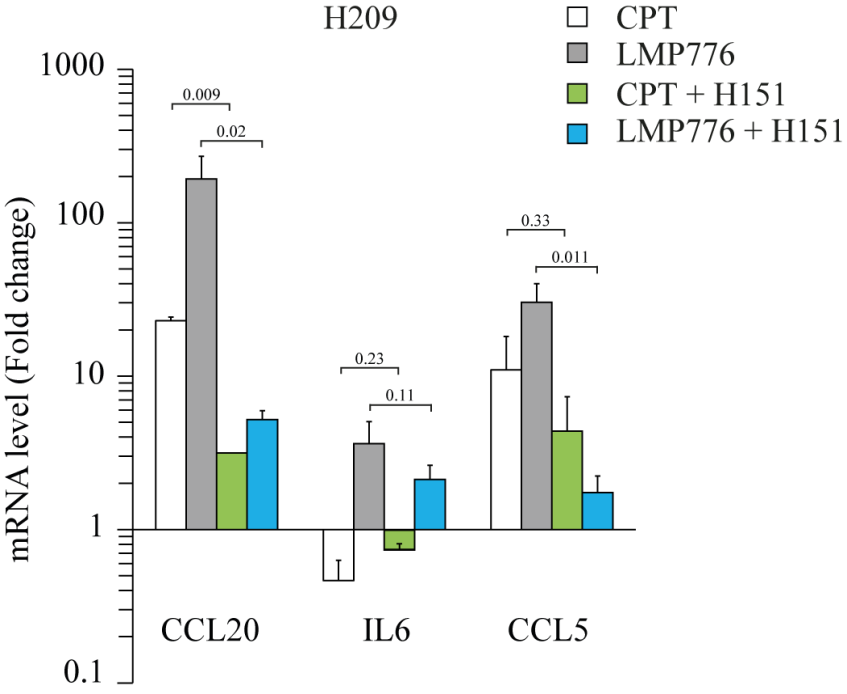
**

**Figure S8. Chemical STING inhibition in H209 cell line.** Expression level of cytokine genes in H209 cell line after H151 administration in response to CPT and LMP776. Data are represented as mean of two biological replicates ± SEM; *p*-values are indicated on the top of each bar and they are calculated as one-tailed ratio paired t-test.

**Supplementary Figure 9.**


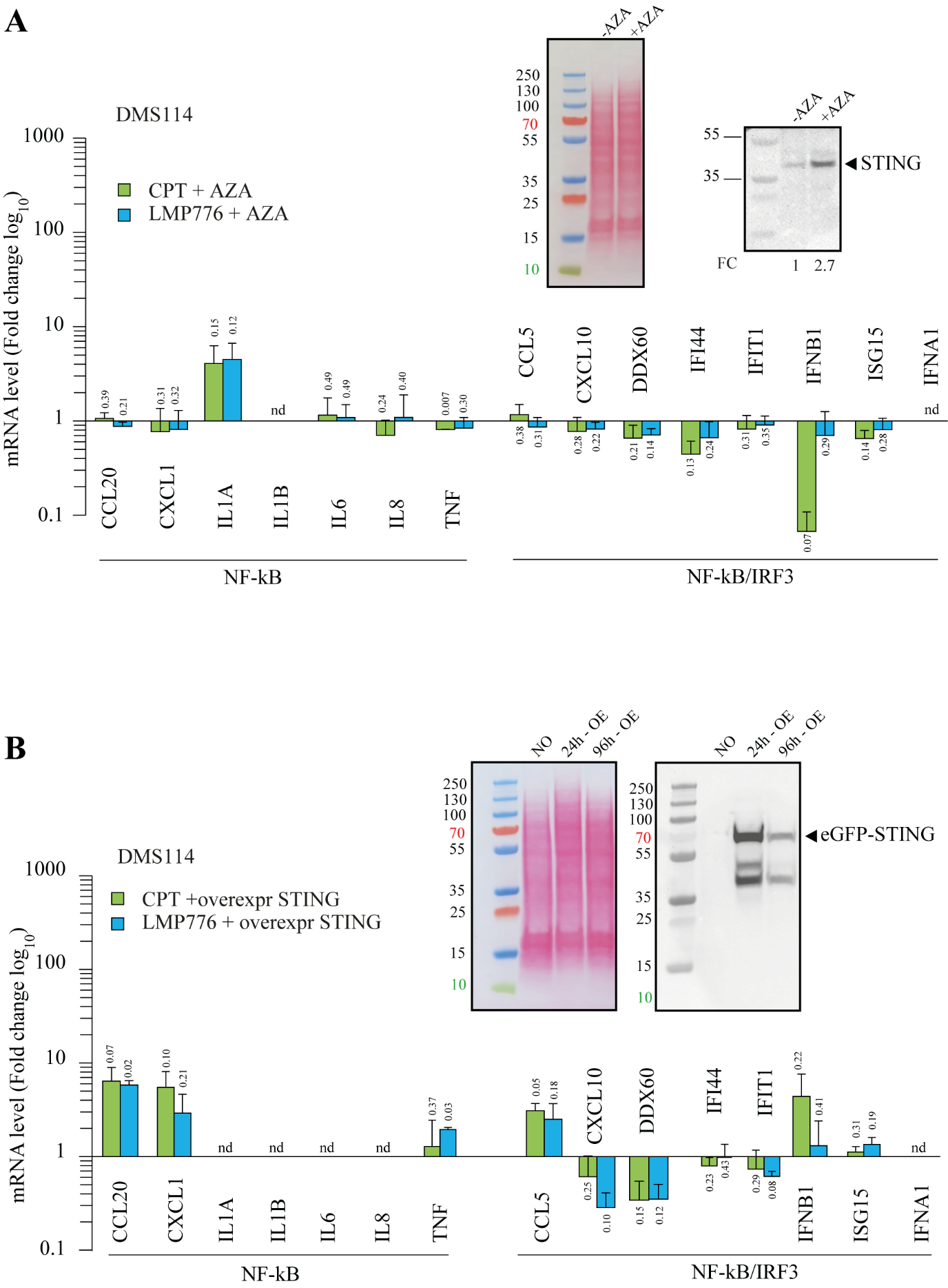


**Figure S9. Induction of STING expression in DMS114. (A)** Expression levels of cytokine genes in DMS114 cell line in response to CPT and LMP776 in presence of 5’-Azacytidine (fold change over non-treated cells in presence of 5’-Azacytidine; nd = not determined value because under detection level). Data are represented as mean of two biological replicates ± SEM; *p*-values are indicated on the top of each bar and they are calculated as one-tailed ratio paired t-test. On the right, WB of DMS114 cell line with and without 5’-Azacitydine. Fold change is calculated over cells non-treated with 5’-Azacitydine. **(B)** Expression level of cytokine genes in DMS114 cell line in response to CPT and LMP776 after STING overexpression (fold change is calculated over non-treated cells overexpressing STING; nd = not determined value because under detection level). Data are represented as mean of two biological replicates ± SEM; *p*-values are indicated on the top of each bar and they are calculated as one-tailed ratio paired t-test. On the right of the panel, WB of DMS114 in tested conditions: non-trasfected (NO), transfected with STING-overexpressing plasmid 24 hous post-transfection (24h - OE), and transfected with STING-overexpressing plasmid at 96 hous post-transfection (96h - OE). The upper band (70 KDa) represents STING protein (42 KDa) conjugated with eGFP (21 KDa), while the lower bands may represent proteolized forms of the protein, or stimulation of endogenous STING expression in transfected cells (42 KDa).

**Supplementary Figure 10**


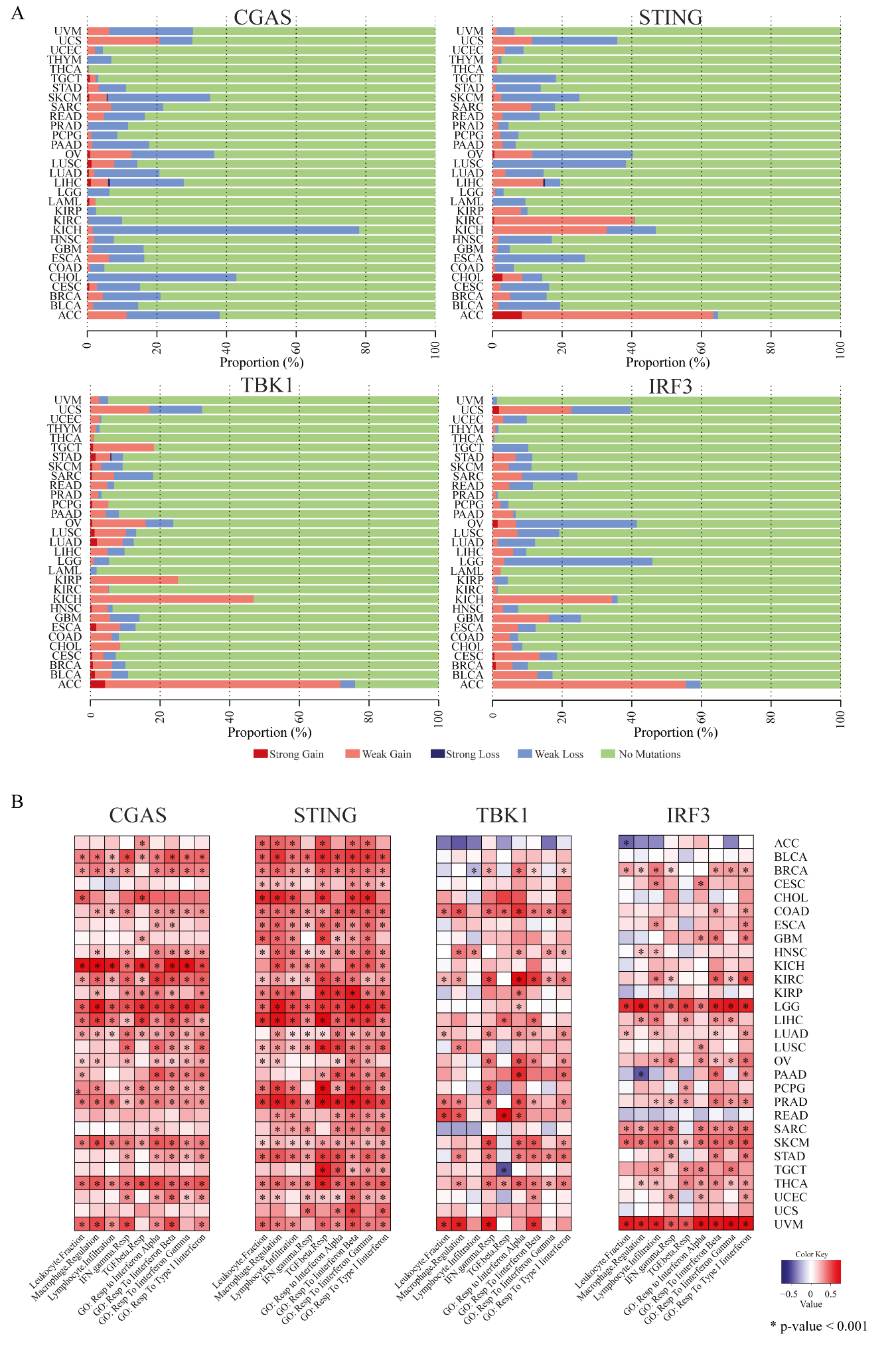


**Figure S10. cGAS/STING Pathway genes are poorly mutated and have a strong correlation with innate immune activation. (A)** Proportion of samples for each cancer type with mutation type as in legend for each CGAS/STING pathway gene. X-axis: proportion (%) of samples. Y-axis: TCGA cancer type. Legend: Strong Gain: copy number variation gain >3; Weak Gain: copy number variation gain =3; Strong Loss: homozygous deletion or heterozygous deletion plus heterozygous damaging mutation or homozygous damaging mutation; Weak Loss: heterozygous deletion or heterozygous damaging mutation. (**B)** Heatmaps showing spearman correlation between gene expression of cGAS, STING, TBK1 and IRF3 and immune signature and enrichment score for Gene Ontology gene sets for each cancer type. Value of correlation is indicated in colour legend. *P*-value of correlation test is reported in the plot.

**Supplementary Figure 11**


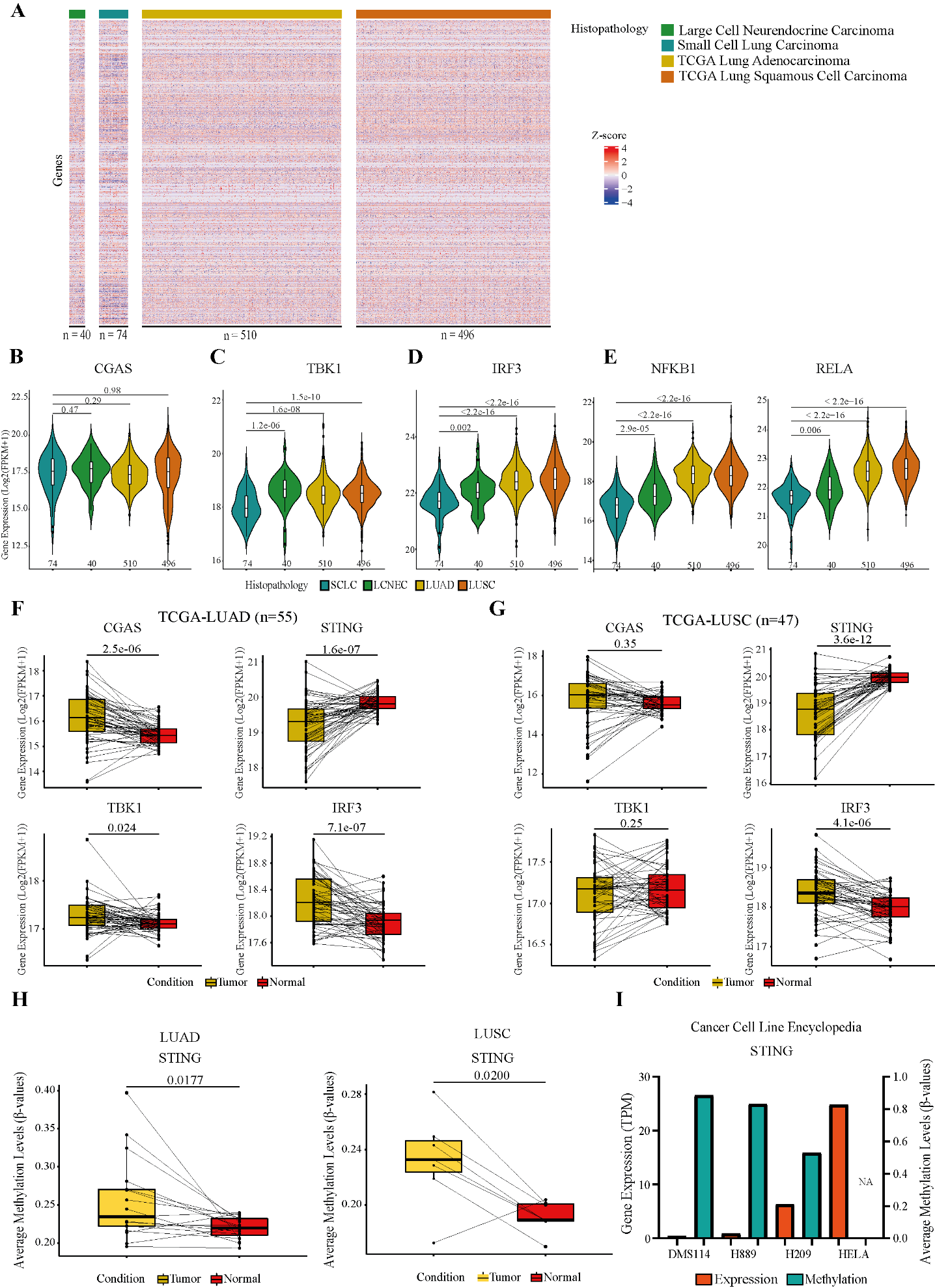


**Figure S11. cGAS/STING pathway gene expression and promoter methylation is impaired in lung tumors and cell lines. (A)** Heatmap of Lung cancer samples analysed in this study. Each column represents a sample and each row the normalized gene expression of each gene. SCLC: Small Cell Lung Carcinoma; LCNEC: Large Cell Neuroendocrine Carcinoma; LUAD: Lung Adeno Carcinoma; LUSC: Lung Squamous Cell Carcinoma. **(B-E)** Violin plot of CGAS, TBK1, IRF3, NFkB1 and RELA gene expression across different lung cancer histopathology types. *P*-value of Wilcoxon test and number of samples for each group are reported in the plot. (**F-G)** Boxplot of cGAS/STING genes expression in tumour and matched normal samples in TCGA-LUAD and TCGA-LUSC cohorts. Lines between boxplots indicates matched samples. Paired-t test *p*-values are reported in the plot. (**H**) Boxplot of methylation levels expressed as the mean of β-values relative to the CpG sites associated to STING gene (y axis) in tumour and matched normal samples (x axis) in in TCGA-LUAD and TCGA-LUSC cohorts. Paired-t test *p*-values are reported in the plot. (**I**) Cancer Cell Line Encyclopaedia (CCLE) gene expression (orange bars) and methylation (light blue bars) data of DMS114, H889, H209 and HeLa cells. For HeLa cell line, data about methylation levels are not available.

**Supplementary Figure 12**


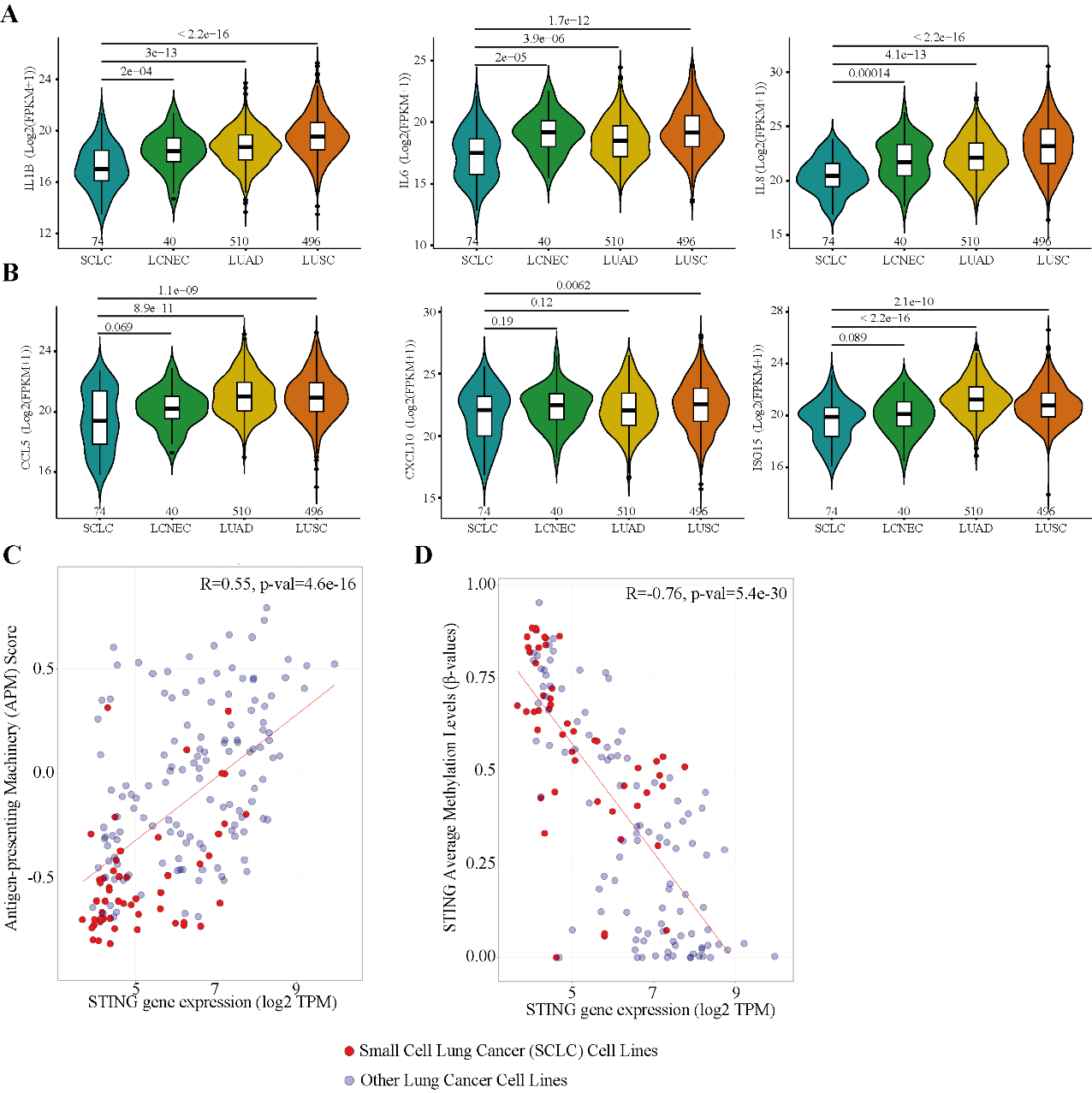


**Figure S12. Cytokine gene expression is downregulated in SCLC tumors and STING impairment correlates with innate immune activation in SCLC cell lines. (A)** Violin plot of representative NFkB1-induced gene expression across different lung cancer histopathology types. *P*-value of Wilcoxon test and number of samples for each group are reported in the plot. **(B)** Violin plot of representative NFkB1/IRF3-induced gene expression across different lung cancer histopathology types. *P*-value of Wilcoxon test and number of samples for each group are reported in the plot. (**C**) Cellminer scatter plot of STING gene expression (x-axis) correlated to “Antigen-Presenting Machinery score. Each dot represents a cancer cell line. Pearson correlation coefficient (R) and *p*-value of correlation test are reported. (**D**) Cellminer scatter plot of STING gene expression (x-axis) correlated to STING gene average methylation level. Each dot represents a lung cancer cell line. SCLC cancer cell lines are highlighted in red. Pearson correlation coefficient (R) and *p*-value of correlation test are reported.

**Supplementary table 1**

| **Gene target** | **Type** | **Sequence** |
| --- | --- | --- |
| IFIT1 Human | Bio-Rad validated primer  460680247; qHsaCED0034841 | |
|  |  |  |
| DDX60 Human | Bio-Rad validated primer  460680232; qHsaCID0006241 | |
|  |  |  |
| CCL5 Human | Bio-Rad validated primer  460680239; qHsaCID0011644 | |
|  |  |  |
| CXCL10 Human | Bio-Rad validated primer  460680254; qHsaCED0046619 | |
|  |  |  |
| IL6 Human | Bio-Rad validated primer  460680246; qHsaCID0020314 | |
|  |  |  |
| CCL20 Human | Bio-Rad validated primer  460680233; qHsaCID0011773 | |
|  |  |  |
| CytB Human | Bio-Rad validated primer  460680241; qHsaCED0048354 | |
|  |  |  |
| TNF Human | Bio-Rad validated primer  460680240; qHsaCED0037461 | |
|  |  |  |
| IL1B Human | Bio-Rad validated primer  4606800238; qHsaCID0022272 | |
|  |  |  |
| IL1A Human | Bio-Rad validated primer  460680243; qHsaCID0016254 | |
|  |  |  |
| IL8 Human | Bio-Rad validated primer  460680259; qHsaCED0046633 | |
|  |  |  |
| ISG15 Human | Bio-Rad validated primer  460680253; qHsaCED0001967 | |
|  |  |  |
| IFI44 Human | Bio-Rad validated primer  460680258; qHsaCED0044799 | |
|  |  |  |
| IFNA1 Human | Bio-Rad validated primer  460680251; qHsaCED0048248 | |
|  |  |  |
| IFNB1 Human | Bio-Rad validated primer  460680231; qHsaCED0046851 | |
|  |  |  |
| CXCL1 Human | Bio-Rad validated primer  460680252; qHsaCED0046130 | |
|  |  |  |
| CCL5 Mouse | FOR | TGCTCCAATCTTGCAGTCGT |
|  | REV | TCTTCTCTGGGTTGGCACAC |
|  |  |  |
| CXCL10 Mouse | FOR | CCAAGTGCTGCCGTCATTTT |
|  | REV | AGCTTCCCTATGGCCCTCAT |
|  |  |  |
| IFIT1 Mouse | FOR1 | TGCTCTGCTGAAAACCCAGA |
|  | REV1 | AGGAACTGGACCTGCTCTGA |
|  |  |  |
| IFI44 Mouse | FOR | TACCCATGACCCACTGCTGA |
|  | REV | ATCAGATCCAGGCTATCCACG |
|  |  |  |
| ISG15 Mouse | FOR | GACCTAGAGCTAGAGCCTGC |
|  | REV | ACCAGGAAATCGTTACCCCC |
|  |  |  |
| CytB Mouse | FOR | ATTCCTTCATGTCGGACGAG |
|  | REV | ACTGAGAAGCCCCCTCAAAT |
|  |  |  |
| cGAS #1 Human | FOR | TGCACGAGTGTTGGAATATTCT |
|  | REV | GAGAAGTTGAAGCTCAGCCG |
|  |  |  |
| cGAS #2 Human | FOR | AAAGAAGGCAGTTTTCACATGAT |
|  | REV | ACCCAAGCATGCAAAGGAAG |
|  |  |  |
| STING Human | FOR | GCAGTTTATCCAGGAAGCG |
|  | REV | AAGGGAATTTCAACGTGGCC |

**Table S1**. **Primers used for qPCR.**

**Supplementary Materials and Methods**

**Cell lines and treatment.** The cancer cell lines HeLa (RRID:CVCL_0030) and U2OS (RRID:CVCL_0042) were purchased from ATCC (LGC Standards S.r.l., Milan, Italy) and were grown in DMEM medium with 10% Fetal Bovine Serum (GIBCO, Thermo Fisher, Waltham, MA, USA) and 2 mM of L-Glutamine. SCLC cell lines H209, H889 and DMS114 have been kindly provided by Anish Thomas (NCI, NIH)[3] and were grown in RPMI medium with 10% Fetal Bovine Serum (GIBCO, Thermo Fisher, Waltham, MA, USA), 2 mM of L-Glutamine, and Pen/Strep 100 µg/mL. Cells were maintained at 37°C in a humidified incubator containing 20% O_2_ and 5% CO_2_. Cell line identity was routinely certified with Cell ID System (Promega, Madison, WI, USA) by BMR Genomics S.r.l (Padova, Italy). Exponentially-growing cells were exposed to TOP1 poisons for the indicated time and concentrations. In case of co-treatments, cells were pre-incubated with Flavopiridol (1μM, #F3055 Sigma), or MG132(R) (25 μM, #SML1135 Sigma) for 1 hour before addition of TOP1 poisons.

An U2OS-derived cell line stably overexpressing RNaseH1 was obtained as reported already [4]. Then, cells were maintained in DMEM culture medium with 10% Fetal Bovine Serum, 2 mM of L-Glutamine, Hygromycin B 500 μg/ml, puromycin 1.5 μg/ml, Penicillin-Streptomycin 100 μg/ml each. For induction, 2 μg/ml of doxycycline was added to culture medium for 48 hours before other treatments were performed.

**Purification of S9.6 antibody.** S9.6 antibody was purified as previously published [4]. Briefly, 800 ml of HB-8730 cellular supernatant was loaded in two columns respectively filled with 2 ml of Sepharose Protein A binding column, (GE Healthcare Chicago, IL, USA) and 2 ml of Sepharose Cl-4B protecting column, (GE Healthcare, Chicago, IL, USA), assembled one over the other. The columns were washed with phosphate buffer pH 8.0 and then the antibody was released from column with C-buffer pH 3.7 (68.8 mM citric acid, 35.9 mM Tris-Na-citrate), collecting the drops into Eppendorf tubes filled with 250 μl of Tris 1 M pH 8.5. After concentration and buffer exchange of positive fractions, the S9.6 antibody was quantified by Lowry Assay and checked by titration in immunofluorescence experiments to obtain the optimal concentration to be used.

**Immunofluorescence microscopy for S9.6 and γH2AX detection (S139-phosphorylation).** Human HeLa cells were seeded at low density (2x10^5^ cells for each 24x24 mm cover glass in a 35 mm dish) and treated with drugs 24 hours after seeding. To stop treatments and fix cells, the culture medium was replaced with ice-cold methanol and incubated 10 minutes at room temperature. Methanol was then removed and cells washed twice with PBS 1X. Acetone was then added for one minutes on ice to permeabilize cells and, after removal of the solvent, cells were washed three times (5 minutes each) with cold PBS 1X under gentle rocking. At this step, glass slides can be conserved at 4°C covered with PBS 1X for few days. Cells were then incubated for 30 minutes at room temperature in Blocking Buffer (saline-sodium citrate Buffer 4X, 0.1% tween20, 3% BSA) under gentle rocking, before incubation with primary antibody for R-loop (S9.6 antibody, 6.25 μg per slide), nucleolin (anti-nucleolin antibody, Abcam Cat# ab22758, RRID:AB_776878, diluted 1:1000) and γ-H2AX (Anti-phospho-Histone H2A.X (Ser139) antibody, Millipore Cat# 05-636, RRID:AB_309864, diluted 1:1000) for 2 hours at room temperature. The incubation with antibodies (both primary and secondary) was performed placing the glass slide upside down on a parafilm slice where a 50-60 μl drop of antibody solution was previously placed. After incubation with primary antibodies, glass slides were washed three times (5 minutes each under gentle rocking) with saline-sodium citrate Buffer 4X (SSC 4X) and then incubated with secondary antibody Alexa Fluor 594 (Goat anti-Mouse IgG Secondary Antibody, Thermo Fisher Scientific Cat# A-11032, RRID:AB_2534091) or Alexa Fluor 488 (Goat anti-Rabbit IgG Secondary Antibody, Thermo Fisher Scientific Cat# A-11008, RRID:AB_143165) for 1 hour at room temperature as explained before. Cells were then washed again three times (5 minutes each under gently rocking) with SSC 4X and incubated with DAPI (3.3 µg/ml in water) for 30 minutes. Glass slides were then mounted upside down in presence of Mowiol on microscope slides. Images were acquired by Nikon Eclipse 90i Microscope (RRID:SCR_020335). Fluorescence quantification analysis were performed using ImageJ software (RRID:SCR_003070). For R-loops, quantification was performed isolating the signal of nucleoplasm from the nucleolus, considering the nucleolin staining of each cell. As nucleolin staining was used to visualize nucleolus only, the acquisition gain of this marker was changed from sample to sample to optimize nucleolus localization in each cell, and thus signal intensity changes are not a measure of nucleolus functional activity. Single cell fluorescence was normalized to the mean of the respective control sample.

For the U2OS-derived cells stably overexpressing RNaseH1, 3x10^4^ cells were seeded on a 24x24mm cover glass in a 35 mm dish and incubated in complete medium without Hygromycin B and Puromycin antibiotics. Twenty-four hours after seeding RNaseH1 expression was induced by adding 2μg/ml of doxycycline to the culture medium for 48h hours. For R-loops detection, cells were incubated the last day of induction with 10 μM of LMP-776 for five or ten minutes. Fixation and immunofluorescence procedures were performed as described above. For γ-H2AX and FLAG detection, cells were treated with 10 μM of CPT and LMP-776 at the indicated time. After induction, cells were fixed and immunofluorescence was performed as previously described [5]. Briefly, cells were fixed 15 minutes with 4% formaldehyde in PBS 1X, permeabilized with 0.25% Triton™ X-100 in PBS 1X for 15 minutes, washed three times 5 minutes each with PBS 1X and blocked with 2% BSA in PBS 1X for 1 hour at room temperature. Slides were co-incubated in blocking buffer overnight at 4°C with the following primary antibodies: Anti-DYKDDDK Antibody (Cell Signaling Technology Cat# 2368, RRID:AB_2217020), diluted 1:800 and anti-γ-H2AX (Anti-phospho-Histone H2A.X (Ser139) antibody, Millipore Cat# 05-636, RRID:AB_309864), diluted 1:1000. The day after, cells were washed three times (5 minutes each) with PBS 1X and incubated in blocking buffer 1 hour at room temperature with the following secondary antibodies: Alexa Fluor 594 (Goat anti-Mouse IgG Secondary Antibody, Thermo Fisher Scientific Cat# A-11032, RRID:AB_2534091), Alexa Fluor 488 (Goat anti-Rabbit IgG Secondary Antibody, Thermo Fisher Scientific Cat# A-11008, RRID:AB_143165). Next, DAPI staining and mounting on microscope slides with Mowiol was performed.

**Immunofluorescence on cGAS and STING.** Fluorescence signals were determined using cells in a coverslip. For STING, cells were fixed with 4% of paraformaldehyde for 10 minutes at RT and then incubated for 1 hour at RT with 1% of BSA, 10 % FBS, 0.1% glycine and 0.1 % Tween-20 under gentle shaking. Then, cells were stained with 0.5 μg/μl of anti-STING antibody (TMEM173 Antibody, Abcam Cat# ab92605, RRID:AB_10562137) overnight at 4°C. For cGAS, cells were fixed with 4% of paraformaldehyde for 20 minutes at RT, permeabilized with 0.5% Triton X-100 in PBS for 5 minutes at RT and then blocked with 1% BSA in PBS for 30 min at RT. Cells were then incubated with anti-cGAS antibody (cGAS (D1D3G) antibody, Cell Signaling Technology Cat# 15102, RRID:AB_2732795) for 1 hour at RT. For both proteins, cells were finally incubated with Alexa Fluor 488 anti-rabbit IgG (Thermo Fisher Scientific Cat# A-11008, RRID:AB_143165) and stained with DAPI (2 μg/μL) for 20 minutes.

**Micronuclei detection by Immunofluorescence.** Adherent cell lines were seeded onto a 6-well plate in presence of 26x26 mm glass slide (2x10^5^ cells each well) and treated the day after with 100 nM CPT or 200 nM LMP776 for 24 hours. After drug removal, cells were left to recover for 48 hours and then fixed for 15 minutes with PFA 4% at RT, permeabilized with Triton 0.5% in PBS for 15 minutes at RT and DAPI stained. Differently, cell lines in suspension were treated at cell density around 7x10^4^ cell/mL with the same protocol but, 48 hours after the end of treatments, 2x10^5^ cells were cytospinned using Cytospin 4 (Thermo Shandon, Runcorn, UK) onto a 26x76 mm glass slide and then fixed, permeabilized, and DAPI stained as for adherent cells. Several images of each glass slide were taken by Nikon Eclipse 90i Microscope (RRID:SCR_020335) to gain a statistically significant number of counted cells.

**RNA Extraction and retrotranscription.** Cytokines expression level was evaluated 48 hours after drug removal (drug concentration 100 nM CPT and 200 nM LMP776). Total RNA was extracted with TRIzol (Ambion, Life technologies, Carlsbad, CA, USA) as manufacturer’s instructions, quantified by UV absorbance and quality controlled by electrophoresis. RNA was converted to cDNA using Superscript III reverse Transcriptase (Invitrogen, ThermoFisher, Waltham, MA, USA) in presence of oligo-deoxythymidines, random hexamers and dNTPs.

**qRT-PCR.** We tested our cDNAs for a panel of cytokines; cDNA was amplified in Biorad CFX Connect Real-Time System by using SsoAdvanced Universal SYBR Green Supermix (#1725274, Bio-Rad, Hercules, CA, USA) and a set of validated primers from each gene locus (Bio-Rad; complete list in table S.1). Amplification protocol was set according to manufacturer’s instructions. Specificity of PCR products was routinely controlled by melting curve analysis and agarose gel electrophoresis. ΔΔCt comparison method was used in order to calculate genes fold change and each gene expression was normalized on CytB.

**Western Blot**. From 30 to 60 mg of total protein lysates were loaded on precast Bolt 4 to 12% Bis-Tris Mini Protein Gel (ThermoFisher Scientific, Waltham, MA, USA). After protein transfer on nitrocellulose, membrane were blocked for one hour at room temperature with blocking buffer BB (TBS 1X, 0,5% tween, 5% milk). The following primary antibodies were incubated overnight at 4°C in BB: TOP1 (Santa Cruz Biotechnology Cat# sc-5342, RRID:AB_2205741, dilution 1:500); STING (Abcam Cat# ab92605, RRID:AB_10562137, dilution 1:1000); cGAS (cGAS (D1D3G) antibody, Cell Signaling Technology Cat# 15102, RRID:AB_2732795, dilution 1:1000).

The day after, membranes were washed three times with TBST (TBS 1X, tween 0,5%) and incubated in blocking buffer with anti-rabbit HRP-conjugated (Abcam Cat# ab205718, RRID:AB_2819160, dilution 1:10,000) or anti-goat HRP-conjugated (Santa Cruz Biotechnology Cat# sc-2922, RRID:AB_656965, dilution 1:2,000). Band detection was performed using Pierce ECL Plus Western Blotting Substrate (ThermoFisher Scientific, Waltham, MA, USA) and Scanner Storm 840 (Amersham Biosciences, Sunnyvale, CA, USA).

**STING gene silencing.** During seeding, Hela cells were transfected with Lipofectamine RNAImax Transfection Reagent (Invitrogen, Thermo Fisher Scientific, Waltham, MA, USA) and 20 nM siRNA against STING (Ambion siRNA #1 128591, Ambion siRNA #2 128592). Protein expression level was monitored against time from 24 to 96 hours post-transfection by Western Blot. All drug treatment were performed 48 hours after transfection and cells were re-transfected with siRNA against Sting after drug removal (72 hours after first silencing).

**RNAseH1 overexpression.** 5x10^5^ HeLa cells/well were seeded in a 6-well plate. 24 after seeding they were transfected with 2.5 µg/well of plasmid for RNAseH1 overexpression (pRH1, gently furnished by F. Chedin, University of California, DAVIS), and 5 µL/well of Lipofectamine 2000 (Invitrogen, Thermo Fisher Scientific, Waltham, MA, USA) in OPTIMEM (Gibco, Thermo Fisher Scientific). Medium was replaced 24 hours after transfection and cells were treated for 24 hours with 100 nM CPT or 200 nM LMP776. Cells were left to recover for 48 hours after drug removal and RNA is extracted as previously reported in this section.

**STING overexpression.** 10^6^ DMS114 cells were seeded in a 6-well plate. 24 hours after seeding, cells were transfected with 2.5 µg/well of plasmid for STING overexpression (NET23 pEGFP-N2- 1174; plasmid #62037 Addgene, Watertown, MA; USA) and 5 µL/well of Lipofectamine 2000 (Invitrogen, Thermo Fisher Scientific, Waltham, MA, USA) in OPTIMEM (Gibco, Thermo Fisher Scientific). Medium was replaced 24 hours after transfection and cells were treated for 24 hours with 100 nM CPT or 200 nM LMP776. Cells were left to recover for 48 hours after drug removal and RNA is extracted as previously reported in this section.

**DNA demethylation with 5’-Azacytidine.** 3x10^6^ DMS114 cells were seeded in a 10 cm dish. The following day, 5 µM 5’-Azacitydine (Cat. A2385 Sigma-Aldrich; Darmstadt, Germany; freshly prepared stock solution 10 mM in water) was added to cells. Because 5’-Azacitydine is highly unstable, daily medium replacements with new 5’-Azacitydine was required. 48 hours after first 5’-Azacitydine administration, cells were treated for 24 hours with 100 nM CPT or 200 nM LMP776. Cells were left to recover for 48 hours after drug removal and RNA is extracted as previously reported in this section.

**Chemical inhibition of STING activity.** 40x10^6^ of H209 cells were seeded in a T-175 flask at a density of 600,000 cells/mL. H151 STING inhibitor at 2 µM concentration (Cat. 6675, Tocris Bioscience; Bristol, UK) was added to flasks and, after 1 hour, 100 nM CPT or 200 nM LMP776 were administered to cells. Cells were treated for 24 hours with 100 nM CPT or 200 nM LMP776. Cells were left to recover for 48 hours after drug removal in presence of H151 (2 µM) and RNA is extracted as previously reported in this section.

**Determination of cellular cGAMP.** Levels of the dinucleotide 2’3’-Cyclic GAMP were measured in whole cell extracts. Cell pellets were resuspended in RIPA buffer (20 mM Tris-HCl, pH 7.5, 150 mM NaCl, 1 mM EDTA, 1 mM EGTA, 1% NP-40, 2 mM DTT, 0.5 mM PMSF and Halt Protease Inhibitor Cocktail), incubated 30 minutes on ice and centrifugated for 20 minutes at 12,000 g at 4 °C. Pellets were then discarded and supernatants were used to determine cGAMP levels with the Direct 2’3’- Cyclic GAMP Enzyme Immunoassay kit (#K067-H1, Arbor Assay).

**Bioinformatic analyses.** Pre-computed data about copy number variations (CNVs), mutations and gene expression were processed and maintained by the Ciccarelli group at The School of Cancer Studies of King's College London and The Francis Crick Institute, as part of the Network of Cancer Genes Database [6]. To compute ratio of mutation for each query gene we used CNVs and mutation data and categorized mutations in 5 classes based on the presence of copy number loss or amplification and of damaging mutations (defined as missense amino acid change as opposed to non-damaging mutations corresponding to nonsense mutations):

• Strong loss mutation: gene with a homozygous CNV loss or heterozygous CNV loss plus
a damaging mutation.

• Weak Loss mutation: gene with a heterozygous CNV loss or a damaging mutation

• Normal: absence of CNVs or mutations

• Weak Gain mutation: gene with a CNV amplification with copy number =3

• Strong Gain mutation: gene with a CNV amplification with copy number >3

Computation of mutation rates and histogram plotting were performed using tidyverse (RRID:SCR_019186) and R base scripts.

To compute correlation between gene expression and enrichment scores, first we computed gene set enrichment score for each tumour sample using ssGSEA function from GSVA library (RRID:SCR_021058). Then, for each query gene and for each cancer type, we computed spearman correlation and produced heatmaps using tidyverse library and R base custom scripts.

For human small-cell lung cancer, mRNA sequencing data (fastq format) and clinical data from EMBL-EGA Data Archive were collected from datasets with accession number EGAD00001001244 (*n=59*) [7] and EGAD00001001431 (*n=15*) [8]*.* For human lung adenocarcinoma (LUAD) and lung squamous cell carcinoma (LUSC) we collected mRNA sequencing data (raw HT-Seq-Counts format, 585 and 550 samples, respectively) and DNA methylation – Illumina Methylation 450k datasets from UCSC Xena Browser. To harmonize SCLC gene expression data with TCGA LUAD and LUSC datasets, we analysed raw RNA-seq libraries using the same mRNA-seq pipeline (Dr15plus version) provided by GDC at https://docs.gdc.cancer.gov/Data/Bioinformatics_Pipelines/ Expression_mRNA_Pipeline/. Human genome version used was the hg38. Genome index for alignment step and gene reference annotation (GENCODE v22, RRID:SCR_014966) were obtained from GDC reference files portal. Gene Set Variation Analyses have been performed using gsva function from GSVA library (v.1.38.1, RRID:SCR_021058) and MSigDB (v.7.2, RRID:SCR_016863) gene sets with default options. Scatter plots and violin plots have been plotted using ggscatter and ggviolin functions from the ggpubr (v.0.4.0, RRID:SCR_021139) library or ggplot2 (RRID:SCR_014601) R package. Data about gene expression an methylation level in cancer cell lines were downloaded from Cancer Cell line Encyclopaedia database [9]. Cellminer Cross Database [10] was used to perform correlation between STING gene expression and STING methylation or antigen-presenting machinery score in CCLE lung cancer cell lines. Gene sets of IRF and NF-kB targets used for ssGSEA analysis were determined by using TRANSFAC database[11].

**Supplementary References**

1. Ohle C, Tesorero R, Schermann G, Dobrev N, Sinning I, Fischer T. Transient RNA-DNA Hybrids Are Required for Efficient Double-Strand Break Repair. Cell. Cell Press; 2016;167:1001-1013.e7.

2. Bou-Nader C, Bothra A, Garboczi DN, Leppla SH, Zhang J. Structural basis of R-loop recognition by the S9.6 monoclonal antibody. Nat Commun. Nature Publishing Group; 2022;13:1–14.

3. Tlemsani C, Pongor L, Elloumi F, Girard L, Huffman KE, Roper N, et al. SCLC-CellMiner: A Resource for Small Cell Lung Cancer Cell Line Genomics and Pharmacology Based on Genomic Signatures. Cell Rep. Cell Press; 2020;33:108296.

4. De Magis A, Manzo SG, Russo M, Marinello J, Morigi R, Sordet O, et al. DNA damage and genome instability by G-quadruplex ligands are mediated by R loops in human cancer cells. Proc Natl Acad Sci U S A. National Academy of Sciences; 2019;116:816–25.

5. Stork CT, Bocek M, Crossley MP, Sollier J, Sanz LA, Chédin F, et al. Co-transcriptional R-loops are the main cause of estrogen-induced DNA damage. Elife. 2016;5:1–21.

6. Repana D, Nulsen J, Dressler L, Bortolomeazzi M, Venkata SK, Tourna A, et al. The Network of Cancer Genes (NCG): A comprehensive catalogue of known and candidate cancer genes from cancer sequencing screens 06 Biological Sciences 0604 Genetics 11 Medical and Health Sciences 1112 Oncology and Carcinogenesis 06 Biological Sciences 060. Genome Biol. BioMed Central; 2019;20:1.

7. George J, Lim JS, Jang SJ, Cun Y, Ozretia L, Kong G, et al. Comprehensive genomic profiles of small cell lung cancer. Nature. Nature Publishing Group; 2015;524:47–53.

8. George J, Walter V, Peifer M, Alexandrov LB, Seidel D, Leenders F, et al. Integrative genomic profiling of large-cell neuroendocrine carcinomas reveals distinct subtypes of high-grade neuroendocrine lung tumors. Nat Commun. Nature Publishing Group; 2018;9:1048.

9. Ghandi M, Huang FW, Jané-Valbuena J, Kryukov G V., Lo CC, McDonald ER, et al. Next-generation characterization of the Cancer Cell Line Encyclopedia. Nature. Nature Publishing Group; 2019;569:503–8.

10. Luna A, Elloumi F, Varma S, Wang Y, Rajapakse VN, Aladjem MI, et al. CellMiner Cross-Database (CellMinerCDB) version 1.2: Exploration of patient-derived cancer cell line pharmacogenomics. Nucleic Acids Res. Oxford Academic; 2021;49:D1083–93.

11. Matys V, Kel-Margoulis O V., Fricke E, Liebich I, Land S, Barre-Dirrie A, et al. TRANSFAC and its module TRANSCompel: transcriptional gene regulation in eukaryotes. Nucleic Acids Res. Oxford Academic; 2006;34:D108–10.
